# A Review on Secure Authentication Mechanisms for Mobile Security

**DOI:** 10.3390/s25030700

**Published:** 2025-01-24

**Authors:** Syed Shabih Ul Hasan, Anwar Ghani, Ali Daud, Habib Akbar, Muhammad Faizan Khan

**Affiliations:** 1 Department of Information Technology, The University of Haripur, Haripur 22620, Pakistan; shabih@uoh.edu.pk (S.S.U.H.); habibakbar@uoh.edu.pk (H.A.); 2 Department of Computer Science, International Islamic University Islamabad, Islamabad 44000, Pakistan; 3 Big Data Research Center, Department of Computer Engineering, Jeju National University, Jeju-si 63243, Republic of Korea; 4 Faculty of Resilience, Rabdan Academy, Abu Dhabi 114646, United Arab Emirates; alimsdb@gmail.com; 5 School of Computer Science & Cyber Engineering, Guangzhou University, Guangzhou 510006, China

**Keywords:** authentication schemes, secure passwords, threats, password attacks, cybersecurity, mobile security

## Abstract

Cybersecurity, complimenting authentication, has become the backbone of the Internet of Things. In the authentication process, the word authentication is of the utmost importance, as it is the door through which both Mr. Right Guy and Mr. Wrong Guy can pass. It is the key to opening the most important and secure accounts worldwide. When authentication is complete, surely there will be passwords. Passwords are a brain-confusing option for the user to choose when making an account during the registration/sign-up process. Providing reliable, effective, and privacy-preserving authentication for individuals in mobile networks is challenging due to user mobility, many attack vectors, and resource-constrained devices. This review paper explores the transformation and modern mobile authentication schemes, categorizing them into password, graphical, behavioral, keystroke, biometric, touchscreen, color, and gaze-based methodologies. It aims to examine the strengths and limitations focused on challenges like security and usability. Standard datasets and performance evaluation measures are also discussed. Finally, research gaps and future directions in this essential and emerging area of research are discussed.

## 1. Introduction

**Figure 1:**
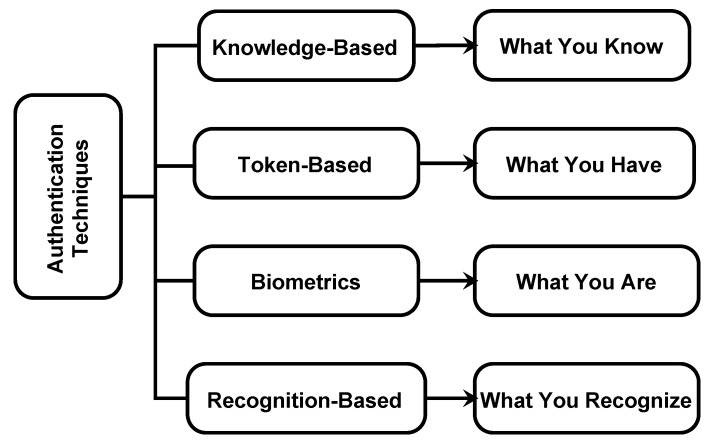
MPAS techniques.

With the emergence of the IoT, mobile password authentication schemes (MPASs) have become much more critical in cybersecurity. Mobiles and tablets are generally utilized to perform basic security and protection exercises, such as versatile ways to save money, portable medicinal services, versatile shopping, etc. Smartphones will soon be able to support applications across a broad range of domains, including home care, social networks, healthcare, environmental monitoring, protection, and e-commerce, thanks to 5G technology, which offers continuous and secure connectivity [1]. With the advancement in technology, the attacking schemes and adversary options have also been widened for hacking, cracking, or guessing a password to gain illegal access to someone else’s account [2]. Many techniques have been introduced to tackle attacks, such as shoulder surfing, dictionary attacks, brute force, etc. Patrick, Long, and Flinn [3] distinguish three security regions for which human factor issues are essential: authentication (passwords), security activities (interruption detection), and creating secure frameworks (building up the security). MPAS is the mechanism for verifying whether someone or something is who or what they claim to be. While being the first line for the protection of the security, the management of user’s access authentication is required to be strong to precisely recognize all types of camouflage behaviors and comprehend the detection of illegal or venomous objects [4]. MPAS can be divided into four types as depicted in (Figure 1).

Security is at its best when it satisfies the sole aim of securing computing atmospheres with the least amount of endangering usability. The ease of use for security has been the main motive of all researchers and scholars in the corresponding fields. The ease of the user in the security field binds all aspects related to human beings and intellectual sciences [5]. Passwords were proved and designed to be the most widely used security mechanism and to have the strength to withstand any attack by an adversary. Security is the door through which the user passes and performs all the tasks after entering an authenticated account. A man spends the least time unlocking the door when entering the office and performs much more work inside than outside.

So, it should be noted that the door-unlocking process, known as security, should be the easiest part of the job. The ease of use should be one of the main aspects of security issues [6,7]. While giving solutions and making the system for securing assets, there should be feedback and review for the users who are using this system to make it more potent by knowing the leakages through suggestions. This evolving process should be performed so relevant users can learn about these improvements. The MPAS should be unpredictable to reduce the risk of exploitation by different authentication attacks [8].

According to mobile market statistics, mobile industry and shipments had overwhelmed the personal computers industry in 2011, and the number of smartphone users also outstripped desktop computer users in 2014 [9]. People are used to accessing their web accounts through mobile devices. Usually, a PIN code or pattern lock unlocks cell phones. Passwords/PIN/pattern locks are exposed to risky environments, even if they are complex and secure. People use easy passwords/pattern locks because it is easy to recall them. Mobile device security is crucial and must be adequate to protect users and their confidential and sensitive data [10]. A secure textual password scheme should protect against different password attacks. Some attacks, like shoulder surfing and smudge attacks, do not need extra software to steal passwords. Shoulder surfing is a significant threat when users access their devices in public places [11]. A shoulder surfer can notice/observe which letters were pressed or what pattern was followed to unlock the device. In less than a minute, a security team at global firms can crack about 80% of employees’ credentials using a network password cracker [12].

Even though smartphones are becoming more popular, several input techniques are available for users who are deficient in communicating with them. Since the availability of MPAS choices is limited, mobile device manufacturers must be more innovative in incorporating MPAS into their devices. Since attackers continuously refine their attack methods, MPAS must be strengthened and updated over time [13].

Several efforts have been made to study MPAS challenges. Douglas et al. Kunda and Chishimba [14] reviewed the MPAS and discussed different authentication protocols, android mobile security, and threats. The cost, protection, privacy, and the simplicity of the MPAS are the four obstacles to implementing strong authentication. More conventional MPASs, such as graphical pattern-based and password/pin authentication, provide medium security at the lowest cost. To overcome the problems of traditional passwords, graphical authentication systems were introduced to provide a strong defense against smudge and shoulder surfing attacks. The authors in [15] reviewed various graphical password techniques. They used different methods to discuss the benefits and drawbacks of graphical password protection. This review also provides a road map for potential changes to various graphical MPAS.

Biometric recognition is a promising area for researchers, but it is plagued by privacy and security issues [16]. A comprehensive study was carried out by Rui and Yan [17] on biometric MPAS. They analyzed the risks of biometric MPAS and suggested several practical and privacy-preserving authentication requirements. The current biometric MPAS was further evaluated regarding its advantages and disadvantages. They found that almost all biometric systems lack biological information for user privacy protection. An important research topic worth addressing is securing users’ private biometric information; this taxonomy indicates a foreign entity (third party) that cannot be relied on.

Ometov et al. [18] summarized the current MPAS regarding usability problems and the main challenges in the current multi-factor authentication systems. Adaptive authentication enables a system, based on factors such as area, connectivity to devices, and other characteristics, to dynamically choose the finest technique(s) for user authentication. To recognize the major open challenges, Arias-Cabarcos et al. [19] reviewed the adaptive authentication literature using design concepts well known in the adaptive systems discipline. Aris and Yaakob [20] addressed the findings of an organized literature review to understand better the present situation regarding the mobile screen locking strategies concerning shoulder surf attacks. A total of 84 techniques were obtained, with 50 non-biometrics. Ten strategies to avoid shoulder surfing attacks were discovered from the fifty non-biometric approaches, which can be used alone or in conjunction with other techniques.

Yuan et al. [21] examined 107 papers using a multilevel taxonomy that included protection and adaptation factors. Conversely, this taxonomy only addresses confidentiality, reliability, and efficiency security objectives. The security aspect concerning privacy preservation. Tziakouris et al. [22] conducted a review with a somewhat different and more focused emphasis. They looked at how the underlying architectures of the published research could be applied to open and ultra-large environments by reviewing the literature on adaptive security. Moving beyond traditional username and password authentication systems has proved difficult. Rittenhouse and Chaudhry [23] discussed alternative authentication schemes besides the username and password mechanism used in Korea. It is critical to avoid replacing a poor authentication scheme with one that is just as bad (or worse) while looking for a replacement (or more possibly additional) authentication scheme. Researchers discussed and included information on how the implementation is commonly used in Korea. Also, they proposed a complex authentication system for the Korean banking environment for secure money transactions from one bank to another. The authors in Ferrag et al. [24] examined a variety of MPASs in depth. They began by providing an overview of current reviewed articles on mobile device privacy published in recent years. The threat models for smart mobile devices were then classified into five categories. This review uses two MPASs, i.e., biometric and number-based (PIN) authentication. A comparison in terms of performance and the limitation of each scheme is presented in tabular form.

This is the first effort to provide a detailed review of almost all the MPASs, including information on how they implemented significant models and their advantages and disadvantages.

The key contributions of this review paper include the following:

- This study examines and categorizes multiple hazards to users’ credentials, focusing on imperfections in traditional password systems and the possible risks to mobile authentication security.
- It categorizes and investigates various authentication algorithms presented by researchers to secure password systems, providing a complete evaluation of methods to mitigate mobile device assaults.
- It explores standard datasets and performance evaluation measures, offering insights into how different authentication methods perform under various conditions and against attacks.
- The study identifies research gaps in authentication systems, highlighting areas where current methods are insufficient and more research is required.
- A comprehensive discussion on challenges to improve the protection of touchscreen smartphones has been addressed in previous studies [25,26]; however, a new viewpoint is identified in this study for potential research directions.
- It studies future research directions, focusing on fostering creativity and creating more robust, secure, and efficient mobile authentication techniques.

Section 2 discusses different password attacks on authentication systems. Section 3 describes different MPASs and their limitations. Section 4 describes the inclusion and exclusion criteria of articles for this survey. Section 5 presents a review of various authentication schemes. Section 6 discusses the performance evaluation and the available datasets for the MPAS. Section 7 presents research questions and relevant future directions, while Section 8 concludes the article.

## 2. Password Attacks

Users should be enlightened and aware of keeping their end of the deal by securing their data. This rule applies to researchers and developers and keeps the system at the top security level [27]. For example, shoulder surfers are able to attack at the time of registration and when logging into a system, which makes the system vulnerable. One should know that the attacker knows what you know and what you propose, as well as what the weakest links in your system are that cannot be kept hidden for a long time from many adversaries worldwide. Problems with passwords are that strong passwords are hard to remember, and easy passwords are easy to remember but are under threat to different attacks [28]. Password attacks that can harm the MPAS are as follows.

### 2.1. Shoulder Surfing Attacks

**Figure 2:**
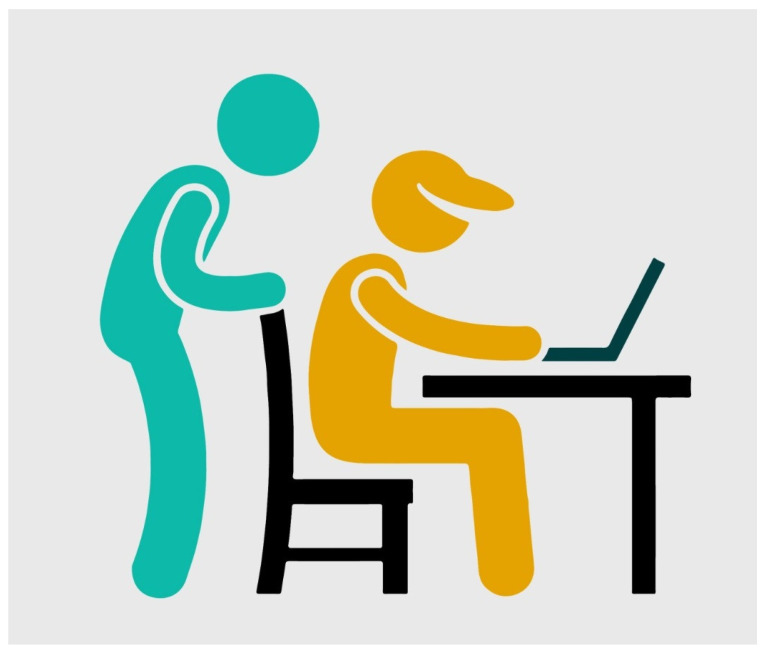
A shoulder surfing situation in a cafe Eiband et al. [29].

Shoulder surfing is the latest weapon hackers use, through direct observations such as looking over someone’s shoulder [29] (Figure 2), or recording their login or other information using a hidden camera. This attack effectively steals the authentication data, i.e., passwords or PINs, as it requires no additional knowledge [30]. Shoulder surfing is mostly pragmatic for spiteful insiders, such as friends and colleagues [2,31]. A Google Scholar search of “shoulder surfing” yields 8420 results (February 2024), with 3600 published since 2017. Most research on shoulder surfing attacks has concentrated on minimizing them during authentication (e.g., password entry) using enhanced authentication schemes or obfuscation approaches. In the last year, 70 of the 92 papers published focused on improved authentication systems [32].

### 2.2. Brute Force Attacks

This attack uses every combination of passwords to break the authentication system. Almost every password or encryption key can be broken down using a brute force attack. The time it takes to brute force into a system is a method to evaluate its security level [33].

### 2.3. Dictionary Attack

A dictionary attack is faster than brute force attacks; rather than trying all the possibilities, it is used to catch the passwords with the most common words used by the user in daily life, i.e., the name of a favorite actress, mobile numbers, etc. [34]. Dictionary attacks can easily judge these passwords. This type of attack is limited to exact matches, but somehow, it is successful since users prefer relatively short passwords that are easy to remember [35,36].

### 2.4. Replay Attacks

Replay attack is also known as reflection attack [37]. It occurs when a hacker detects secure network communication, intercepts it, and then resends it (or “replays” it) as if it were their own. This attack class involves the data unit’s leak and transmission to obtain an unauthorized effect or reuse the message to cheat others [38,39].

### 2.5. Phishing Attacks

The hacker redirects users to the fake website for their authentication details [40]. For example, if a user desires to open a website that says www.facebook.com, the user is diverted to a different website by the attackers, i.e., www.faceboOk.com, having the same interface as the original, and the user enters credentials without knowing that they have been tricked. The hacker or attacker steals the user’s login information and redirects them to a fake information website. Phishing frauds have been gaining attention, as these attacks have increased numbers [41].

### 2.6. Key Loggers

Key logger (v.11.6.23 24 October 2024) is a computer software program that records user activities in various ways, i.e., screen, voice, keyboard, mouse, and keyboard logging in invisible mode [42]. The attacker installs the key logger software on the user’s computer system; the software creates a log file that keeps track and sends the log file to the attacker’s email address with all the user’s pressed keys, through which they can obtain the login data and can access the important files [43].

### 2.7. Guessing Attacks

Guessing attacks always remain a severe threat [44,45]. Here, an attacker performs repeated login trials by guessing the possible values of the user’s password. CAPTCHAs [46] may be a good defense aid for graphical and text passwords.

### 2.8. Smudge Attacks

**Figure 3:**
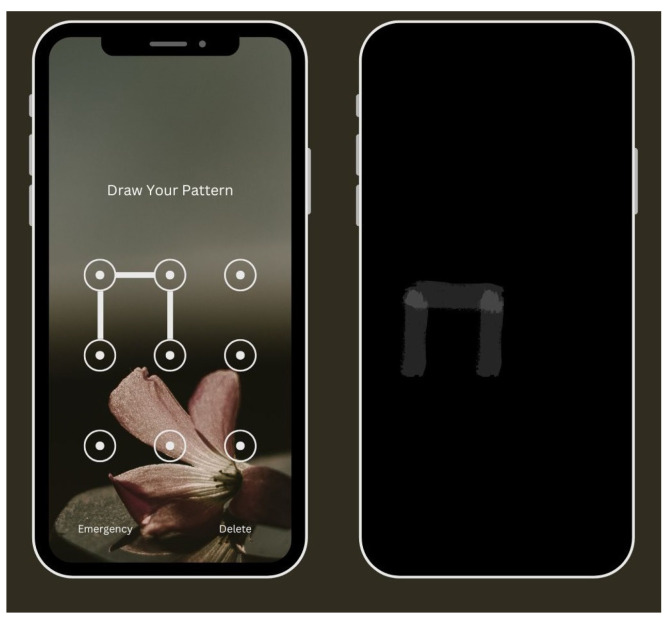
Smudge of finger on mobile [48].

To unlock a cell phone, the user draws a retained unlock pattern with a finger on the touchscreen, where the finger leaves its slick buildups, called smudge, shown in Figure 3 on the touchscreen’s surface. An unauthorized user can abuse the smudge to recreate the mystery design [47].

### 2.9. Electroencephalography (EEG) Signal

An intruder might try to gain access to the mobile phone by drawing the pattern of the user trying to imitate the user’s electroencephalography (EEG) signal. It is performed with the headset of EEG; the pattern is drawn, and the machine is deceived into allowing mobile device access [49]. Using EEG as a biometric attribute has significant advantages over traditional methods. Authenticating a system requires a predetermined mental state that cannot be forced. Non-living brains cannot create EEG signals, making it impossible to bypass authentication systems by assassinating users. This authentication mechanism protects both the asset and the users. The EEG features make it appropriate for new technologies like IoT [50].

Table 1 illustrates attacks on mobile touchscreen devices.

## 3. Recent Security Reviews

The threat models and defenses of the 2FA mobile money scheme were reviewed in [25]. To review the pertinent literature, the writers employed a suitable search method. This review study showed that security vulnerabilities must be fixed because the existing mobile money 2FA technique only uses a subscriber identity module (SIM) and a personal identification number (PIN) for user authentication, which are vulnerable to attacks. Mayrhofer and Sigg [53] focused on the adversary models for mobile device authentication

Transparent authentication techniques for mobile device security were thoroughly analyzed and reviewed in [26]. This study highlighted the need to examine the application’s sensitivity level and determine whether or not a particular application needs to be protected when authenticating the mobile user.

Aslam et al. [54] discussed the evaluation of authentication protocols that are necessary to gain access to telecare medical information systems and covered their advantages and disadvantages concerning computing costs, security, and privacy. One-factor, two-factor, and three-factor authentication techniques are separated into three main groups. To determine which authentication approaches and methods are most effective in various scenarios, Velásquez et al. presented them in [55]. Kilinc and Yanik [56] examined and ranked several authentication and key agreement protocols based on their security and performance characteristics.

With an emphasis on Android platforms, Faruki et al.’s survey from [57] included many security-related topics, including the strengths and weaknesses of well-known malware analysis and detection and code modification techniques. After examining many malware samples and various approaches to combating the diverse new malware, they concluded that a thorough evaluation framework with strong static and dynamic methodologies might answer this new issue.

Meng et al. [58] extensively investigated biometric-based techniques for mobile phone authentication because passwords and PINs are authentication solutions with numerous disadvantages. The authors of the survey study examined the viability of implementing both physiological and behavioral approaches on touch-enabled mobile phones, identified potential attack spots, and suggested countermeasures. Their investigation led them to conclude that the system’s security and usability can be improved by implementing a hybrid authentication mechanism combining standard PINs or passwords with multimodal biometric authentication.

Multimodal biometrics-based fusion methods have been determined to be the most efficient regarding security and usability, using physiological and behavioral biometrics-based procedures similar to those studied in [58] continually and not only during initial access. One major problem with biometric features is the potential for theft, which can be avoided using template protection measures. In 2016, a comparable survey [59] was published covering touch dynamics authentication methods for mobile devices. Under the umbrella of behavioral biometrics, touch dynamics records how a user interacts with a touchscreen device for static and dynamic user authentication.

Password managers enable users to store and access credentials securely across devices, decreasing password repetition. While desktop and browser-based password managers have been extensively researched for security flaws, mobile password managers have received less attention. Sharma and Mishra [60] looked at three popular Android password managers and evaluated their password generation, vault security, and autofill features. The findings showed flaws, such as insecure password generation, unencrypted metadata storage, and phishing concerns. There are recommendations for upgrading mobile password managers and the Android operating system and suggestions for future security research.

Only a few survey articles mentioned above addressed mobile device authentication systems; none detailed their features. To the best of our knowledge, this study is the first to fully address the features of security analysis methodologies, security systems, authentication schemes, threat models, and countermeasures that the research community has recently offered. Table 2 summarizes the recent relevant survey papers.

## 4. Article Selection Criteria

This systematic review utilizes significant scholarly resources from Web of Science (WoS), IEEE: Piscataway, NJ, USA, Elsevier, and ScienceDirect databases through a comprehensive search method. These databases are chosen for their extensive coverage and authoritative content, providing access to high-quality academic research across multiple fields. Our research focuses on relevant literature from 2018 and 2024, using keywords like “secure authentication”, “presentation attacks”, “biometric security”, “graphical passwords”, “gaze authentication”, “fingerprint detection”, and “countermeasure technologies”, among others. These terms are intentionally blended to yield the most relevant and intriguing findings. The study we conduct not only covers recent technological advancements but also identifies gaps and promising areas for future research. This contributes to the continuing discussion about improving MPAS.

### Inclusion and Exclusion Criteria

Our selection criteria focus on research that directly addresses our key subject of MPAS and exclude unrelated or inadequate studies. Our rigorous literature selection has yielded a detailed overview of authentication approaches, highlighting current obstacles and possible future advances. The focus is on research that provides data from experiments on MPAS effectiveness against the presentation of different attacks. Articles addressed in the literature have undergone mandatory peer review to ensure they satisfy scholarly standards. Those articles covering the technological aspects of authentication without considering its attacks on the methodology are excluded. Non-peer-reviewed sources such as articles of opinion and commercial publications are excluded.

## 5. Authentication Schemes

**Figure 4:**
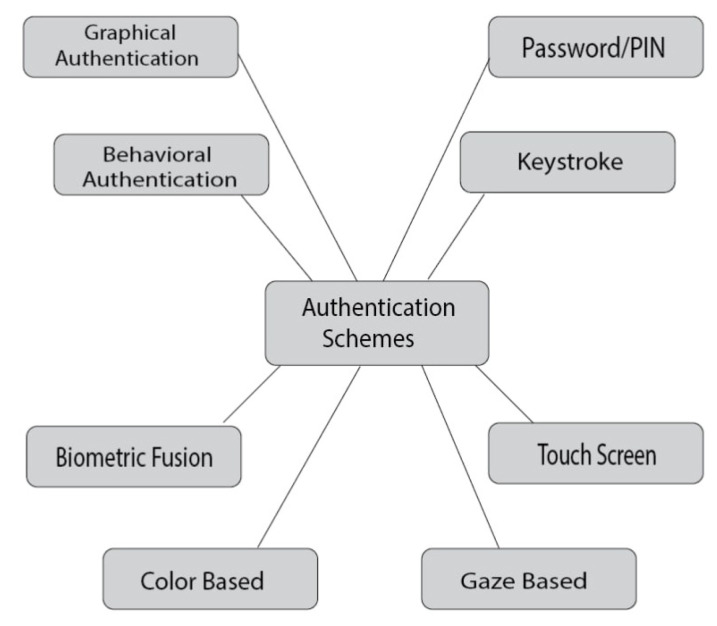
Authentication schemes.

Different researchers used various techniques to ensure secure MPAS. Some of them are discussed in this section (Figure 4).

### 5.1. Password/PIN and Pattern Authentication

Text passwords are still common because they offer several benefits. They are simple to learn, enforce, and adjust if they are corrupted or forgotten, and they are incredibly reliable. Unfortunately, the widespread use of text passwords across thousands of modern user accounts has made creating and remembering a unique and random text password for each account cognitively impossible [61].

PINs were first utilized in automated dispensing and control systems at gas stations. They were later introduced to “the Chubb system” by the Westminster Bank in the United Kingdom in 1967 [62]. Since then, PINs have become increasingly common in the banking business worldwide. PINs operated as passwords to protect embedded devices (such as PDAs and smartphones) from unwanted access, thanks to the rapid development of microelectronic technology in the 1990s. The idea of employing passwords to imitate human choices of four-digit PINs was initially proposed by Bonneau et al. [63], and it has sparked a slew of new PIN research [64].

The classic PIN-entry method is commonly used but is subject to attacks such as shoulder surfing and spyware since it requires direct input. Binbeshr et al. [65] presented an indirect input method that uses addition mod ten and a mini-challenge keypad to produce a one-time PIN (OTP) while concealing the original PIN. Their user study demonstrates that this strategy improves security over traditional PIN systems while maintaining usability, with user comments supporting its use in high-security contexts.

The DRAW-A-PIN authentication scheme is a suggested way of further improving the PIN system [66]. In this authentication scheme, a user must draw the PIN code on their mobile screen instead of simply typing out their PIN code. When a PIN is drawn, the authentication system will verify the digits entered and then observe the user’s behavior during the PIN entry.

Authentication based on patterns is also a common form of authentication today on many mobile devices [67]. Graphical pattern passwords are preferred by many users due to the human brain’s preference for visual material over letters and numbers Ye et al. [68]. Pattern unlock is quite vulnerable to attacks like shoulder surfing and smudge attacks. People choose simple and easy PIN/passwords and patterns to authenticate because these schemes are easy to use and available on almost every mobile device.

Alajmi et al. [69] proposed a CAPTCHA AI hard problem-based salted challenge–response MPAS. The proposed framework is based on the same principle as CAPTCHA. i.e., a bot’s ability to recognize twisted text in a picture is a complex problem. Using a client’s password, the framework combines the challenge text. It scatters it within a random image rather than submitting it in a way that humans find easy but bots find prohibitively tricky.

#### 5.1.1. Opportunity

It is not necessary to use the direct password input technique. In social engineering attack scenarios, the attacker can observe the user’s behavior, including password entry operations, while the user is operating their mobile device. All types of display information, including user guide material, should be masked against new attack types if the methods entail direct password input procedures. The best approach to achieve this is to create a password entry method that is not direct. When using indirect input, guessing the information from the shown data is challenging. As a result, regular PIN codes can be combined with an indirect input mechanism to allow personal identification and authentication.

#### 5.1.2. Entropy in Security Usability Tradeoffs

The entropy offered by PINs is limited, which limits their usage for applications having less security; on the other hand, the entropy offered by passwords is high and provides robust security. With the help of policies and enhancements, security has improved over time but at the cost of usability. Providing balance is the aim of modern approaches like two-factor authentication, biometrics, and adaptive authentication. Table 3 summarizes the comparison of PIN and password authentication schemes in terms of entropy and usability.

### 5.2. Authentication Using Behavioral Features

Soon, smartphones will likely include intelligent access control systems allowing continuous authentication. Behavioral authentication and behavior modeling are closely related techniques. There is an extensive study on behavior modeling approaches to anticipate user characteristics such as traits, personality, and behavior [70].

Researchers have proposed using behavioral touch MPAS as second line of defense if initial authentication is compromised [71,72], or as the center for the user who does not configure any authentication techniques due to usability issues [73]. Behavioral authentication is a form of biometric authentication with two benefits: first, it is implicit, ensuring it is performed unconsciously. Second, behavioral characteristics are challenging to mimic because it is difficult for others to learn and replicate a person’s behavioral habits after they have been established [74]. Behavioral biometric information, i.e., touch gestures, mouse movements, and keystrokes, can be gathered via sensor devices and help analyze the user’s behavioral attributes for authentication [75,76]. In government departments, passport offices, border surveillance, and many consumer devices, there is a growing need for privacy-preserving biometric authentication systems [77].

A biometric device can be divided into two categories based on the number of modalities used: unimodal and multimodal. Unimodal biometric systems focus on a single identifier depend on a single modality for authentication; therefore, they are easier to create. The authentication metric can be a single point of failure; a unimodal device faces challenges such as noisy data, poor recognition efficiency, less reliable results, and spoofing attacks [78,79]. A multimodal biometric system uses multiple or combined parameters (for example, face and voice features). It does not depend on a single feature, making it much more stable and difficult to break. It is more resistant to spoofing threats, has higher recognition rates, and has improved accuracy and reliability [80].

Machine learning schemes can be used to improve protection schemes for MPAS [81]. Machine learning techniques are highly successful in enhancing the protection of applications for touchscreen mobile devices. The key advantages of mobile phone safety software training algorithms are recognizing biometric and sensor information to improve the security of mobile phones [58].

Bo et al. [82] suggested using a support vector machine (SVM) algorithm to create a classification model based on cell phone users’ biometric behavior. The classification model developed updates the SVM model by introducing new features found through self-learning to enhance classification accuracy. The results show that the proposed authentication scheme for identification is fast and accurate.

Song et al. [83] proposed an authentication framework using multi-touch devices for the use of physiological and behavioral biometrics by the consumer using K-nearest neighbor (KNN) and the support vector machine (SVM) algorithm. The legitimate hand geometry and compartmental knowledge of users were employed to create a one-class SVM and KNN classification model. The experiment showed that, although the proposed system uses a small number of experimental subjects and analyzes a few movements, the KNN outperforms the SVM in nearly every case.

Ehatisham-ul Haq et al. [84] suggested that the performance of the three user-authentication algorithms should be measured and evaluated using Bayesian networks (BNs), SVM, and KNN. The classifiers were trained to create a mobile user MPAS based on their physical behavior. The three classifiers’ precision was compared. The proposed solution cannot provide various access rates when identified based on their biometrics of actions.

Liang et al. [85] proposed authentication scheme using convolutionary neural network (ConvNets) to predict the user tap series and device usage behavior. Sensor data were collected, as users communicate with the system on different applications, and a classification model based on ConvNet, SVM, and KNN was established using sensor data. The solution suggested did not make the CovNet model more complex to obtain stable and better performance.

Sajjad et al. [86] proposed a hybrid technology with two layers of security. The first layer integrates the palm vein, fingerprints, and face recognition, and the second layer takes these things along with face anti-spoofing using a convolutional neural network (CNN)-based model to detect spoofing. After matching fingerprints successfully, it is checked on a CNN-based model to verify that it is fake or real—repetition of the same method with the face and palm. Experimental results verified the efficient work of the system, conquering the constraints in spoofing techniques.

Dynamic Time Wrapping (DTW) was introduced to strengthen the security of mobile devices [87]. For the MPAS, only a tiny number of DTWs were used. DTW was used to build a scheme [66] that validates the user by observing how they draw a PIN on a touchscreen rather than typing it. Based on the user’s PIN drawing behavior, the DTW algorithm was utilized to compare and realize the similarities between the two PIN drawing samples. The experiment’s findings showed that the users’ PIN writing behavior can be used for personal identification, and DTW can support the proposed model with promising results. The proposed research used a small number of experimental subjects and did not equate its findings with those of other classification algorithms.

Current mobile authentication methods that use PINs and physiological biometrics face severe security and usability problems. Stragapede et al. [88] focused on continuous authentication (CA), utilizing behavioral biometrics, which allows for passive user verification without requiring explicit actions. A comparative analysis was undertaken using behavioral attributes gathered from everyday mobile interactions such as typing and tapping and sensor data from numerous mobile sensors. HuMIdb, an innovative public dataset, has significant mobile interaction data that may be used for testing and research in passive authentication. Combining (fusion) many modalities consistently increased authentication accuracy, resulting in a considerable reduction in EER compared to single-modality systems.

Stragapede et al. [89] introduced BehavePassDB, a publicly available mobile behavioral biometrics and benchmark evaluation database. This database enables continuous authentication by exploiting multimodal data from user interactions with mobile devices organized into separate sessions and jobs. The authors presented an experimental protocol that allows for fair comparisons of various approaches while investigating the performance differences between random and skilled impostor scenarios, emphasizing the effectiveness of Long Short-Term Memory (LSTM) architecture with triplet loss in distinguishing user identity from device characteristics.

Sejjari et al. [90] investigated swiping motions as a mechanism for continuous identity verification on mobile devices, demonstrating that behavioral biometrics can be a more user-friendly and effective authentication method than older methods. The study showed that various machine learning classifiers, notably a deep learning model, outperform other models in reliably identifying users based on their distinctive swiping behavior. The study achieved a low Equal Error Rate (EER) of 0.20%, demonstrating hopeful progress in continuous biometric authentication. Table 4 summarizes the behavioral authentication schemes.

#### 5.2.1. Opportunity

Data encryption and profiling procedures should be performed on the server to reduce unnecessary energy consumption. Mobile devices should consume as little energy as possible and should only be utilized to detect or sense the owner’s actions. Then, using algorithmic selection, a subset of the data retrieved from each sensor’s raw data should be sent to the server. The server could profile and encrypt the data for authentication purposes before sending them to the mobile device using the selected data. When the data are received, the mobile device can compare them to the current user’s behavior pattern.

#### 5.2.2. Entropy in Security Usability Tradeoffs

The entropy provided by behavioral authentication systems ranges from moderate to high; the reason behind it is the uniqueness and variability in user behavior. With the enhanced usability due to the passive authentication, these systems face challenges side by side like environmental sensitivity and impersonation attacks. The machine learning hybrids models and adaptive thresholds have evolved, which has increased the usability in terms of security, which makes the behavioral features auspicious for future authentication systems. Table 5 summarizes the comparison of behavioral authentication schemes in terms of entropy and usability.

### 5.3. Keystroke Authentication

For MPAS, keystroke dynamics has advanced and is now used in mobile phones. The main problem with cell phones, however, is that they can be used anywhere. As a result, examining the utilization of keystroke dynamics using data obtained in different typing positions becomes essential [92]. Keystroke elements successfully perform biometric authentication for user validation at a workstation [93]. Several research studies have been performed for MPAS using keystroke techniques [94].

Khan et al. [95] tested the vulnerability of smartphone keystroke dynamics to password stiffening and mimicry attacks. They used feature analysis on a publicly available dataset [96] to create interfaces that teach users to mimic their victim’s keystroke behavior and propose two schemes for an attacker to get real-time guidance when performing a mimicry attack. Against many passwords, their setup effectively circumvents keystroke dynamics. The researchers conducted experiments to demonstrate how malicious insiders can use social engineering to gather keystroke data and then use that data to recreate the victim’s behavior.

Buchoux and Clarke [97] used a keystroke user authentication scheme and designed software to run on Microsoft Windows Mobile 5. They proposed two types of passwords: a strong alphanumeric password and a simple PIN. Three classifiers were also evaluated: Mahalanobis distance, FFMLP, and Euclidean distance. Their results suggested that as the PIN increased the amount of input data, the performance of the defined classifiers was better when the password was employed. People usually use either a PIN or pattern to unlock their cell phones. Users always prefer the simple PIN schemes proposed here because they are short and easy to use.

Saevanee and Bhattarakosol [98] proposed a new mechanism for MPAS, named finger pressure, combined with inter-key features and existing hold time. Users utilize a mobile touchpad as a touchscreen to measure finger pressure. Results showed 99% accuracy, as this system does not require remembering any complex passwords or PINs, just a simple password combined with the user’s behavioral manners.

Zahid et al. [99] examined keystroke data from 25 mobile device users. The proposed mechanism takes a total of six characteristics of the keystroke. These characteristics of various users are scattered, and a finicky classifier is utilized to classify and cluster the data. The proposed system has an error rate of 2%, which indicates that the system is user-friendly and can be adopted.

Hwang et al. [100] suggested MPAS using the keystroke dynamics, which depends on a four-digit PIN. Usually, a four-digit PIN cannot give secure authentication and is vulnerable to guessing and shoulder surfing attacks. The authors introduced an input scheme supported by tempo cues and artificial rhythms to make it more secure. Their experiment showed that the proposed technique reduces the energy efficient ratio from 13% to 4%. Table 6 summarizes the keystroke authentication schemes.

#### 5.3.1. Opportunity

As technology advances, mobile and portable devices are becoming increasingly common in people’s daily lives. Smartphones and tablets have ever-increasing memory and processing power compared to a few years ago. Furthermore, advanced and sensitive microhardware sensors can unlock new feature data, such as multi-touch touchscreens, pressure-sensitive panels, accelerometers, and gyroscopes. This upgraded hardware is now widely available, paving the way for future research into keystroke dynamics on this platform.

#### 5.3.2. Entropy in Security Usability Tradeoffs

The entropy provided by behavioral authentication systems ranges from moderate to high, the reason behind it being the uniqueness and variability in user behavior. With the enhanced usability due to the passive authentication, these systems do face challenges side by side like environmental sensitivity and impersonation attacks. The machine learning hybrid models and adaptive thresholds have evolved, which has increased the usability in terms of security, which makes the behavioral features auspicious for future authentication systems. Table 7 summarizes the comparison of keystroke authentication schemes in terms of entropy and usability.

### 5.4. Touchscreen Authentication

The touch MPAS has been called “the more natural, unobtrusive future of smartphone biometrics”. Google launched pattern lock as a security safety mechanism in 2008 [105].

The authors in Colley et al. [106] developed a touchscreen unlocking technique that improves the attack resistance of the commonly used pattern lock mechanism. Their scheme increases the touch password capacity for a specific smudge pattern by 15 factors. In a user test (*n* = 36), users found the model to be more reliable than their current lock system while still being comparable in speed and memorability. The proposed process took an average of 2.2 s to unlock the phone (SD = 0.9); 1.5 s is the average mean time (SD = 0.6) for patterns with only the multi-select function, comparable to those illustrated for the regular mechanism for pattern locking.

Research by Eiband et al. [107] provided reasonable measurement steps to hide the password or PIN codes by a bystander. Their presented work focused on hiding messaging texts like chat from a shoulder surfer. The researchers thought that text messages in public places should be written in the user’s handwriting since it is not easy for everyone to read other handwritten words. It replaces the standard printing font on mobile devices with user-personal handwriting. The user study showed that there is variation in the times of reading the user’s handwriting and reading the text written in the handwriting of different users.

**Figure 5:**
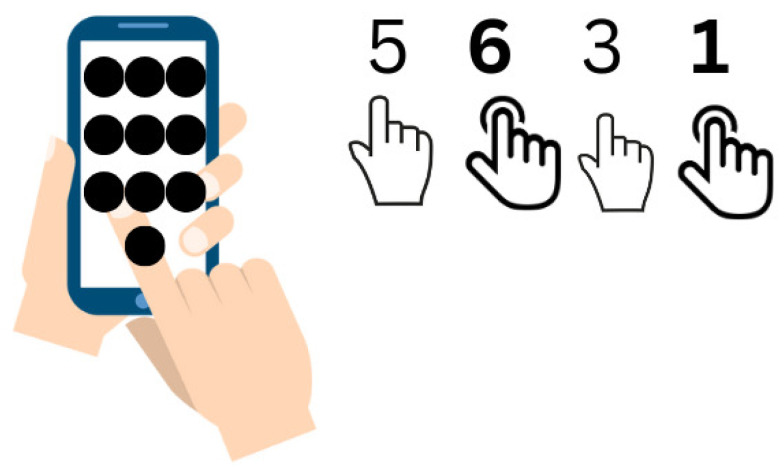
Overview of force PINs Krombholz et al. [108].

Krombholz et al. [108] proposed a technique to avoid password attacks for the MPAS in public areas. The idea is to ensure the security of PINs by applying force to the digits which are either bold or underlined, e.g., 1 **2** 1 **4** as shown in Figure 5. It also gives vibration feedback as the user enters any PIN with force authentication. This scheme is easy and simple, as most users use PIN authentication to log in.

**Figure 6:**
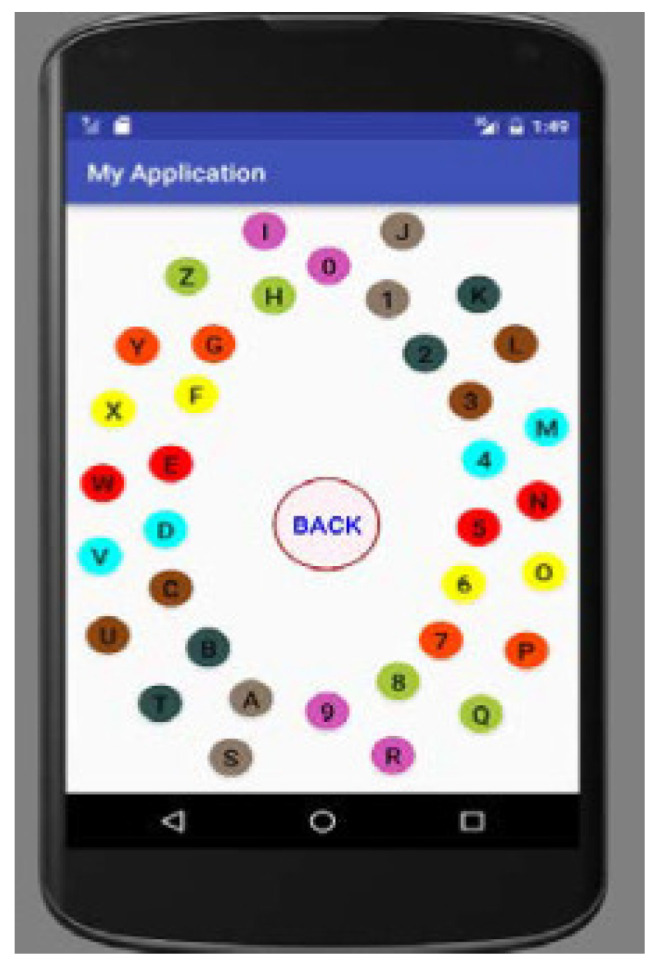
User on-screen interface for smartphone Chakraborty et al. [109].

Chakraborty et al. [109] designed a system that lets the user choose their password. The proposed process was reviewed based on usability and security perspectives compared to existing approaches that can be repeated. MobSecure has 36 nodes that have all alphabets (A–Z) and numeric characters (0,1⋯,9) (Figure 6). They are arranged in a circular pattern named orbit, so there are two orbits, inner and outer. All nodes are colored, and the two adjacent nodes of both orbits are colored the same and named sister nodes. The authentication process consists of two stages. In the first stage, the user is challenged, in which the user can hear a number through earphones, which can be random and consist of numbers between 0 and 17. The second stage entails the user selecting their number from the inner or outer orbit. The proposed process was reviewed based on usability and security perspectives compared to existing approaches.

The 3×3 pattern lock scheme is standard compared to textual and numerical PINs. Due to a lack of instructions on complex drawing rules, users prefer to use fundamental patterns, making them vulnerable to attacks. Vaddepalli et al. [110] proposed a mobile unlocking application that uses a pattern formation with a PassO circular grid layout instead of a 3×3 grid. Using a mobile device, they examined the Passo patterns obtained from 32 participants in a lab study. According to the findings, the average duration of patterns was reduced by 25.47% in the mobile scenario. As a result, participants developed shorter patterns in real-life situations for ease of use and recall.

Gattulli et al. [111] explored a method for continuous user authentication on smartphones by leveraging touch events and human activities. The authors utilized the H-MOG dataset to analyze user behavior while reading documents on a smartphone, focusing on the combination of touch events and sensor data (accelerometer, gyroscope, and magnetometer). They proposed a feature extraction method that includes the Signal Vector Magnitude and evaluates various machine learning models, including 1-class and 2-class SVMs, achieving highly effective application behavioral biometrics in the context of the public health importance of continuous authentication to enhance smartphone security, particularly against unauthorized access during passive activities like reading.

The research by Finnegan et al. [112] suggested a scoping that examines the current state of behavioral biometrics in user authentication and demographic characteristic detection, particularly in measuring screen time on mobile devices. The review systematically analyzed 122 studies that utilized built-in sensors on smartphones and tablets for user authentication through methods such as motion behavior, touch dynamics, and keystroke dynamics. The findings indicated that touch gestures and movement are the most commonly used biometric methods, with a significant reliance on accelerometer and touch data streams. However, the overall quality of the studies was low, highlighting a need for more rigorous research, especially involving child populations, to apply behavioral biometrics in public health contexts effectively.

#### 5.4.1. Opportunity

Possible future work and opportunities in this touchscreen authentication techniques are therefore in the development of increased security, practicality derived from new technologies. Future developments in AI and machine learning can enhance touchscreen authentication and make improvements in analyzing customer patterns, with the possibility of risk-based and better policies advanced with improved detection. As a part of the new challenges, there is a smaller set of input modes to investigate—new types of inputs could be gesture or stylus inputs, for instance—and there is a set of threats that remain urgent topics in the field of adversarial attacks and spoofing. Additional measures will also enhance standardization and user awareness, hence improving the usage of these evolved authentication methods.

Table 8 summarizes the touchscreen authentication schemes.

#### 5.4.2. Entropy in Security Usability Tradeoffs

The entropy provided by touchscreen authentication techniques ranges from moderate to high, which also provides the user-friendly interaction. The vulnerability and environmental sensitivity still cause challenges in terms of attacks like shoulder surfing and smudging. Features like machine learning, hybrids, and behavioral features expansion amplify the strength of security in terms of usability. By balancing the security and usability for modern devices, the touchscreen authentication systems are advancing speedily. Table 9 summarizes the comparison of touchscreen authentication schemes in terms of entropy and usability.

### 5.5. Gaze-Based Authentication Techniques

Gaze is an appealing and vital communication methodology that gives the client an instinctive, without-hand method for connection. Traditional techniques like touch and clicking have usability advantages. Still, the gaze is more protective against observation attacks due to its subtlety. It can be implemented to existing mobile pattern lock schemes with no additional changes to the interface [117]. Gaze data can be used in numerous viewpoints, for example, gadget verification, diversion plan, gadget controlling, client conduct examination, etc. [118]. Gaze as features of input [119] have empowered different look-based verification strategies, in the case of shoulder surfing, that can be grouped crosswise over three common classes [120], i.e., gaze PIN-based, gaze gesture-based, and gaze pursuit-based authentication.

Khamis et al. [121] proposed GazeTouchPass, a multimodal verification conspire in which clients characterize four images; every single one must be typed in, either utilizing touch (a digit somewhere in the range of 0 and 9) or utilizing gaze (looking to one side and one side). Successive look contributions to a similar heading would then be isolated by a gaze to the front. GazeTouchPass accomplishes a harmony between security and convenience, with low confirmation times and high observation resistance.

Katsini et al. [122] developed a two-step process for evaluating the quality of client-made graphical passwords dependent on the eye-gaze behavior during password formation. In the first step, the user gaze patterns are determined, represented by the exceptional obsessions in each area of interest (AOI) and the all-out obsession length per AOI. Second, the gaze-based entropy of the user is determined. A feasibility study was conducted to assess password robustness. Results uncovered a solid positive relationship between the quality of the made passwords and the gaze-based entropy.

Abdrabou et al. [123] presented and evaluated six MPAS using gaze, gestures, and multimodal combinations and found that gaze offers a good balance between utility and protection, is highly protected in the case of shoulder surfing attack, needs minimum time for authentication, and is barely vulnerable to mistakes. Experimental results showed a 70% improvement over previous authentication time work due mainly to improved sensors and visual computing schemes.

Abe and Yamada [124], from the peak velocity of the gaze, proposed a scheme for predicting the target gaze point coordinates. The polynomial approximation of the peak velocity and the distance to the target was used to model the prediction estimation function. Furthermore, this modeling result was experimentally accurate using BioEye 2015’s RAN task data. Moreover, it was demonstrated that this modeling outcome had individual variations, and the application of individual certification was described.

Constantinides et al. [125] proposed an eye gaze-driven metric approach focusing on the hotspot vs. non-hotspot image sections for elegantly measuring the intensity of graphical passwords created by the users by evaluating eye gaze actions of the users during password formation. For testing the feasibility, an eye-tracking study (n = 42) was conducted, i.e., the presence of link within the metric that is proposed and the intensity of passwords created by users, where a graphical password is constructed by the user using a customized image which activates declarative memory (familiar image) vs. an image demonstrating (generic image).

Namnakani et al. [126] introduced GazeCast, a novel system that utilizes users’ handheld mobile devices to facilitate gaze-based interaction with public displays. The authors highlighted the limitations of traditional gaze interaction methods, which often rely on stationary or cumbersome eye trackers. Through a user study involving 20 participants, they compared GazeCast with a standard webcam setup. They found that while GazeCast required more time and physical effort, it offered higher accuracy and flexible positioning. The study demonstrated that GazeCast could enhance the user experience by allowing spontaneous, calibration-free interactions while addressing privacy concerns by enabling local processing of gaze data on users’ devices. They concluded by discussing the potential applications of GazeCast in various public settings and the importance of further research to optimize its usability. Table 10 summarizes the gaze-based authentication schemes.

#### 5.5.1. Opportunity

Gaze-based security solutions require highly accurate gaze estimates to be completely implicit and work without the user’s participation. Calibration is necessary to gather exact gaze data [129]. Eye trackers used to require users to be extremely still, even requiring them to utilize chin rests [130]. While current eye trackers allow users to roam around to some extent, they frequently require recalibration whenever the user’s or setup’s state changes dramatically.

However, calibration adds a layer of complexity to the contact process, making it feel tedious, awkward, and time-consuming [131]. Many studies have made calibration more implicit than explicit, such as including it in the engagement process while reading text or viewing videos. Previous research on implicit calibration focused on generic use cases rather than implicit authentication. It leaves room for future research into how to adjust implicit authentication to improve its performance. It necessitates first comprehending the implicit gaze-based authentication to better trade between the calibration time and accuracy.

#### 5.5.2. Entropy in Security Usability Tradeoffs

The entropy provided by gaze-based authentication ranges from moderate to high, which also provides hands-free, user-friendly interaction. Advancement in features like machine learning, hybrid systems, and dynamic stimuli importantly increased the security along with addressing the challenges related to usability. Hardware requirements and environmental sensitivity are still hurdles in the adoption of these systems. For applications like accessibility-focused ones, gaze-based authentication has the capability to amplify the usability and security in biometric systems. Table 11 summarizes the comparison of gaze-based authentication schemes in terms of entropy and usability.

### 5.6. Graphical Passwords Authentication

Initially, any MPAS necessitates the acceptance of a security system that is simple, versatile, and adaptable. The graphical information-based MPAS is among the schemes of authentication that depend on the remembrance of protected passwords [132]. Researchers have found that graphical passwords are more memorable than textual alphanumeric passwords [133]. Organizations or different social networking websites force users to adopt a strong password policy, which requires users to select hard passwords that are less vulnerable to discovery. Nevertheless, on the other hand, it increases the burden on the users to remember those hard passwords [134]. The user likes to use easy passwords for all systems, raising the security risk, as one attack can compromise all systems [135]. If people choose easy passwords, they are easy to find through automated search [136]. The primary debate regarding graphical passwords is that they reduce the overhead load of user memory to remember hard textual passwords, as studies have proved that humans memorize graphics and images better than text [137]. Because of this memorability advantage, users are keen for graphical secret keys [138].

Recognition-based, pure recall-based, and cued-recall graphical passwords are the three types of graphical passwords [139]. The images selected correctly during registration are recognized in a recognition-based authentication scheme. The procedure, however, is possibly interrupted by phishing attacks that mislead users from capturing screenshots of their passwords. Another drawback to this scheme is the discovery of some pre-selected images that involve scanning several images regarding the password, making the operation time-consuming [140]. Users must recreate or draw something because the password depends on a pure-recall authentication scheme. When a stylus is not used, the disadvantages of schemes depending on recognition are fixed by the schemes depending on pure recall; however, they are vulnerable to misconceptions [134]. Since they automatically mimic human inputs, the systems that rely on pure recall are slightly exposed to social engineering, dictionaries, and brute force rather than text-based passwords. Cued-recall authentication involves the user seeing a specified picture and clicking on one or more predetermined locations in a prescribed sequence. In contrast to the complicated and real-world segment, preconceived click objects need clear, artificial images, such as cartoon-like images. The user is susceptible to selecting the image’s hot spot, which would be easy for a hacker to guess [141].

Graphical passwords are widely used in search of a remedy for shoulder surfing attacks. They are easy-to-remember and hard-to-crack passwords, as pattern recognition of biometric passwords while drawing the graphical passwords has been considered to provide better security [142].

**Figure 7:**
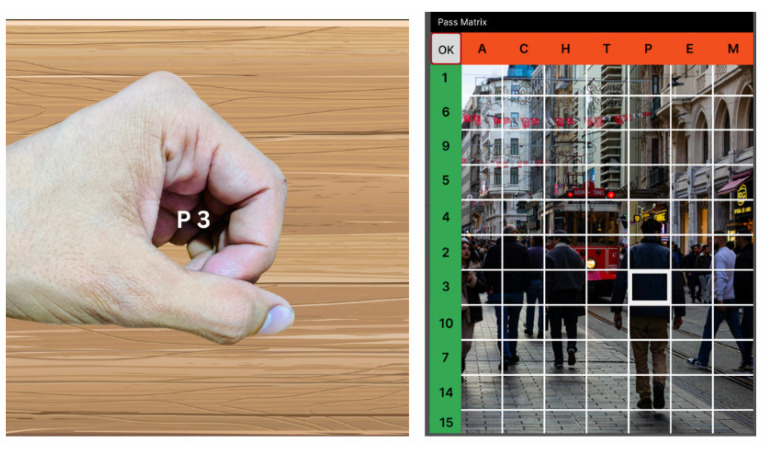
Login indicator, login screen of pass matrix authentication scheme Sun et al. [143].

A technique named pass matrix [143], which consists of four modules, namely, image discretization, horizontal and vertical axis module, login indicator generator module, and a communication module, gives a broad space for a password to the user. This technique avoids shoulder surfing and smudge attacks. The login indicator will generate a new password for every session; the users will use a dynamic pointer to identify the position of their password rather than clicking on the password directly. The user images are divided into a 7×11 grid; the smaller the image, the larger the password space. Each time the user logs in, they will touch the screen to see the indicator, which can also be referred to as a session password shown in Figure 7. The given indicator will be converted into an image with horizontal and vertical axes from A to G, and 1 to 11 characters representing a 7×11 grid, respectively, shown in Figure 7. The module for password verification confirms the arrangement within the pass square for each image. The user can log in to the system if the arrangement is accurate in every image.

Techniques depending on click, known as passport [144], are among the earlier techniques in graphical passwords. Still, in this scheme, security analysis found that its simple geometric pattern with images is vulnerable to hotspots [145,146,147].

**Figure 8:**
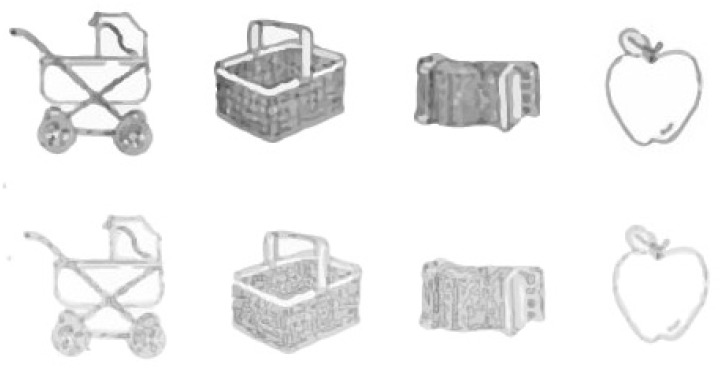
The target image from the degraded version of the target image.

In their research, Cain and Still [148] concentrated on a series of graphics based on faster authentication while choosing the right images in 14s. There are many distracting images from which the user has to touch or click the four target images. This series of images comprises low-grade line drawings of daily life objects. Low-quality or distorted unclear images are used to support cognitive object recognition. The system uses Recognition by Components, which tells us that 3D objects can be recognized without the viewpoint of curving, linearity, shape, etc. These graphics are vague and unclear, making them hardly identifiable objects in Figure 8.

**Figure 9:**
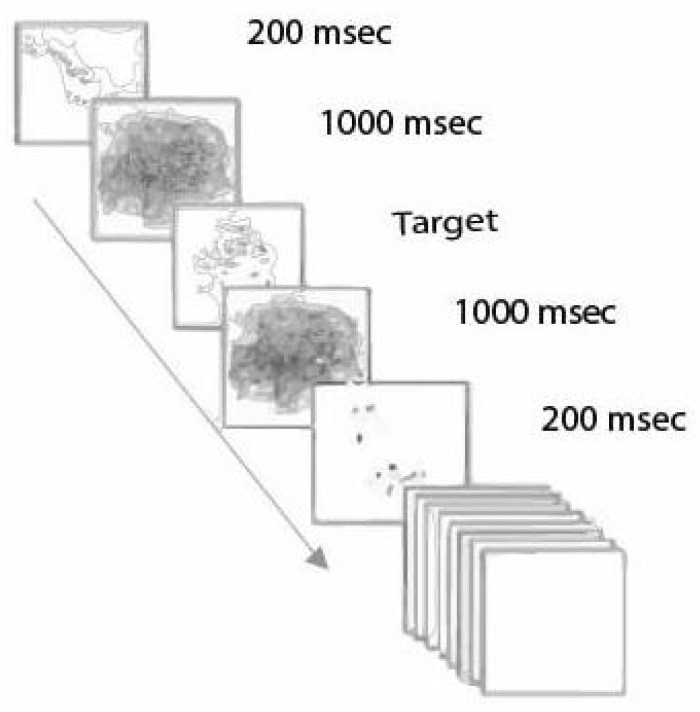
Degraded images and their masks.

At the same time, these nebulous graphics can be quickly recognized if the user is acquainted with the original object. Tainted images are shown on the screen for a fraction of a second, 200 ms to be precise, which is fast, but the authentic user can easily cope with it. The degraded images overlap to create a mask that stops for one second and comes after each degraded image shown in Figure 9. These degraded pictures are displayed randomly in between seven distracting masks. The correct target image is not shown initially to allow the user time to fine-tune the streaming speed.

**Figure 10:**
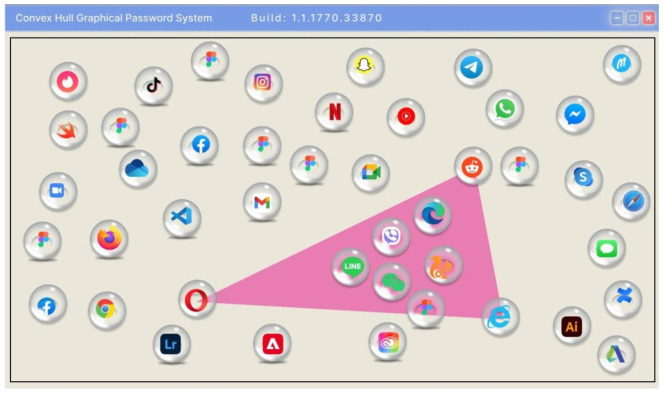
Example of a convex hull with 3 pass-icons Wiedenbeck et al. [149].

A graphical MPAS, known as the Convex Hall Click Scheme [149], is based on a round of image selection for authentication as shown in Figure 10. The convex hall contains pass icons that can be clicked without clicking the user’s actual password icons.

**Figure 11:**
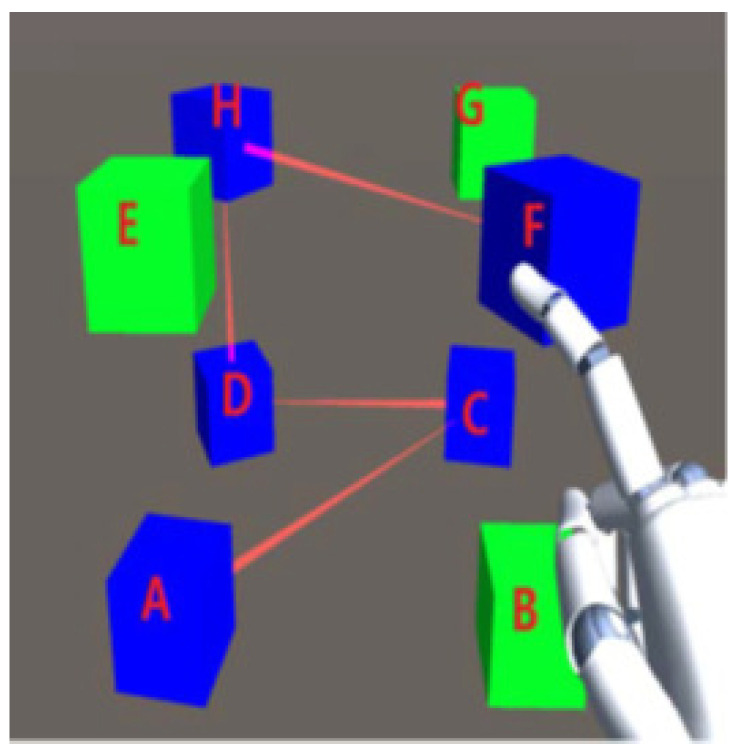
An example of a 3D graphical password created by the user.

Three-dimensional graphical passwords have introduced a new methodology for user authentication on mobile devices. Unity 3D package [150] is used to create the 3D graphical password, and a device named Leap motion, with the help of a scripting tool, c#, allows user interaction with mobile devices. The 3D graphical methodology is shaped like a cube matrix, consisting of nodes and edges that construct the user password. This cube has eight green cubes of the same structure; Leap can see the user’s hand movement. The activity by the user’s hand is seen within the observation area; the 3D virtual hand will represent the user’s hand movement, which permits user interaction with objects as shown in Figure 11. The positions of the cube can be randomly set. When the user touches any cube, its state visibly changes. The arrangement of the being touched is meant to record and pass through an algorithm to create a unique password for every login.

Irfan et al. [151] suggested a graphical password scheme based on text. Out of sixteen random images, the user selects five images. The random images shown are a mixture of graphical and text-based images in password creation. This scheme’s login process consists of a 4×3 grid, in which the last vertical grid shifts continuously. If the selected moving grid image is matched with the other two chosen static grid images, the user can tap the image to activate the unit. The drawback of this scheme is the waiting time for the synchronization of images, which results in increased login time.

The research study titled “Securing Access to the Internet of Medical Things Using a Graphical-Password-Based User Authentication Scheme” by Khan et al. [152] proposed a novel graphical password authentication method aimed at enhancing security in the Internet of Medical Things (IoMT). The authors highlighted the growing need for secure user authentication in digital healthcare services and address the limitations of traditional text-based passwords. The proposed scheme incorporates multiple factors, including simple arithmetic operations, machine learning for hand gesture recognition, and medical images for recall, to create a user-friendly and memorable authentication process. The method’s effectiveness was evaluated using the Post-Study System Usability Questionnaire (PSSUQ), which showed significant improvements in the system, information, and interface quality compared to conventional PIN and pattern-based techniques. The study concluded that the proposed graphical authentication scheme is a promising solution for improving security and usability in IoMT applications.

Table 12 summarizes the graphical authentication schemes.

#### 5.6.1. Opportunity

Graphical elements are usually prominent. As a result, when compared to text-based passwords, graphical passwords may be more vulnerable under certain circumstances. An image is larger than the text, assuming the graphical password using a predefined picture selection method; however, a shoulder surfing attacker may have problems acquiring the original password from a long distance with a text-based password. As a result, a shoulder surfing attacker may obtain the password from a considerable distance. As a result, one or more authentication measures must be used in conjunction with a graphical password.

#### 5.6.2. Entropy in Security Usability Tradeoffs

The graphical passwords entropy governs theoretical security, on the other hand the practical entropy suffers, because of the user behavior and attacks like shoulder surfing and smudges. The evolution of graphical passwords systems happens with time by adding complexity to them which intensifies the security but declines the usability. The key challenge remains the same, the balance between both (security and usability), with the help of adaptive and hybrid systems showing promise for the future. Table 13 summarizes the comparison of graphical passwords authentication in terms of entropy and usability.

### 5.7. Color-Based Authentication

Color MPAS has been proposed as an alternative to textual and graphical image selection passwords. A method for detecting tampered regions on images is color image authentication [155].

Potey et al. [156] proposed a technique whose registration process begins with entering the information submitted by the user, such as username, password, contact number, etc. Then, the user has to choose three colors from the colors grid arbitrarily, and the order of these colors is important, so the user has to remember the order in which they entered the colors. After this user has to click on three shades: white, black, and gray. This order is also to be memorized by the user, which will also be needed at the time of authentication. For authentication, the user must enter their username and then offer the same order of the shade of the three basic shades: white, black, and gray. This is the first step. If correct, the user has to choose the numbers shown on the colors grid, which signify the column number of the corresponding numeral grid. Those three cells have white, black, and gray in the numeral grid. Each corresponding row is recognized per the shade order selected in the registration process. Then, the user has to choose the three-digit numeric present in the numeral grid by applying the same procedure using the identified row. The user has to then append the three numbers from the numeral grid for a single-time created password. Then, the same procedure must be rerun for the second and third colors. After completing nine colors, the submit button is to be pressed, and if all of the combinations and the final digit are correct, authentication is granted.

Jain et al. [157] proposed a system of authentication using graphical and color systems to overcome the attack of shoulder surfing. This system provides security against shoulder surfing and key logger attacks. By combining the color sector and numeric password, the user can easily authenticate to the system.

Chiang and Chiasson [158] proposed a multi-layered drawing unlock scheme that, when compared to pattern unlock, significantly expands the pattern space. More complex patterns can be formed using multiple layers and warp cells at the grid’s corners. When a warp cell is touched while entering a pattern, for example, the second layer grid appears, concealing the authentic grid layer for proceeding with the pattern entry.

Gugenheimer et al. [159] proposed ColorSnakes, a software-based authentication system that provides shield against shoulder attack and, to a minimal level, attacks involving the videos. A ColorSnakes PIN begins with a single colored digit and ends with four digits. Users draw an arc from the first colored digit to their PIN. The users enter their PIN; various colored entrap pathways are intended to be generated simultaneously among the starting colored digits, mimicking the alternative path to conceal the input. The underlying numbers grid is created randomly after each successful input to prevent smudge attacks.

Woods and Silvennoinen [160] introduced a unique solution that uses color as a memory cue to improve password memorability and security. A five-week longitudinal study looked at over 3000 passwords established, learned, and recalled. Their findings indicate that adding color to passwords can improve memorability and security. Allowing users to select their password colors instead of pre-selected ones promotes personalization and important memory cues. Color increased password entropy, adding another layer of protection. These discoveries have practical consequences for academics and practitioners, potentially improving password security and reducing financial damages from breaches.

Selamat et al. [161] proposed an advanced color-based authentication by making the authentication process more difficult, addressing flaws in textual passwords, and improving security at the authentication layer.Table 14 summarizes the color-based authentication schemes.

#### 5.7.1. Opportunity

Some authentication schemes use color combinations that may confuse the user when becoming used to the system. As color-blind people have difficulty identifying colors, the schemes must be user friendly.

#### 5.7.2. Entropy in Security Usability Tradeoffs

The entropy provided by color-based authentication is low because of the minimal predictability and choices. These schemes provide a replacement to traditional methods in the form of user-friendly interaction. With the advancement in features like dynamic palettes, multimodal integration has increased security along with usability. As a user-friendly replacement for secure and accessible authentication, color-based authentication schemes are continuously evolving. Table 15 summarizes the comparison of color-based passwords authentication in terms of entropy and usability.

### 5.8. Processed Authentication and Random Password

**Figure 12:**
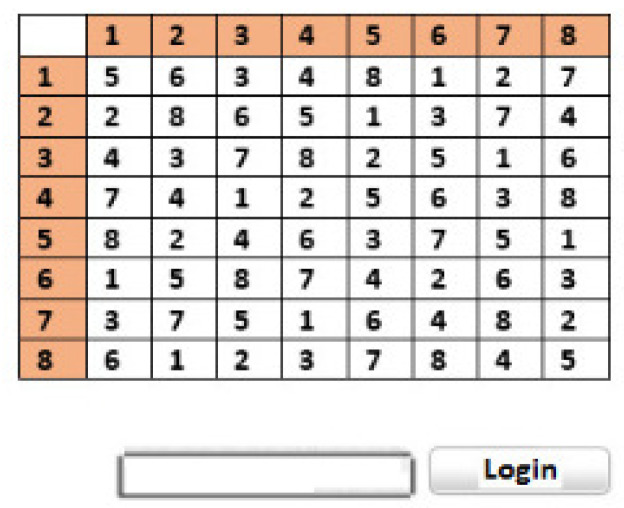
Login process of pair-based authentication system.

Authentication through textual passwords is the most common way to log in. However, it still has many vulnerabilities attached. Prabhu and Shah [131] proposed a system of two methodologies against these attacks. In the first method, pair-based authentication, the user must choose a password of a minimum of eight characters. It is a secret password that will never be entered, but it will help to make and enter the actual password during that session. When the user starts the login process, the system presents a 7×7 grid, shown in Figure 12 with session passwords made according to the hidden password. These session passwords are combinations of alphabets, numbers, special characters, and different symbols, and change every session.

When the grid is shown to the user, the user considers the two letters of their hidden password from the grid, and then the intersection of those two hidden passwords is c, which is the actual session password. These two represent a row and column; their intersection is the letter L, which is the session password the user must enter. The password chosen at the time of registration should be an even number of characters so that each pair can generate an intersecting session password. The second scheme is the hybrid authentication of the user during registration, which includes a group of colors in which they have to select the ratings of the colors. The colors can have similar ratings, which the user has to remember. When the user wants to log in, the system uses an 8×8 grid with random numbers from one to eight. Four pairs of colors are shown to the user. Each pair represents a row and a column. The user considers their rating of the color and then concentrates on the crisscrossing number that intersects after combining the two numbers they obtain from the colors (Figure 12). This number would be the session password the user enters for authentication.

**Figure 13:**
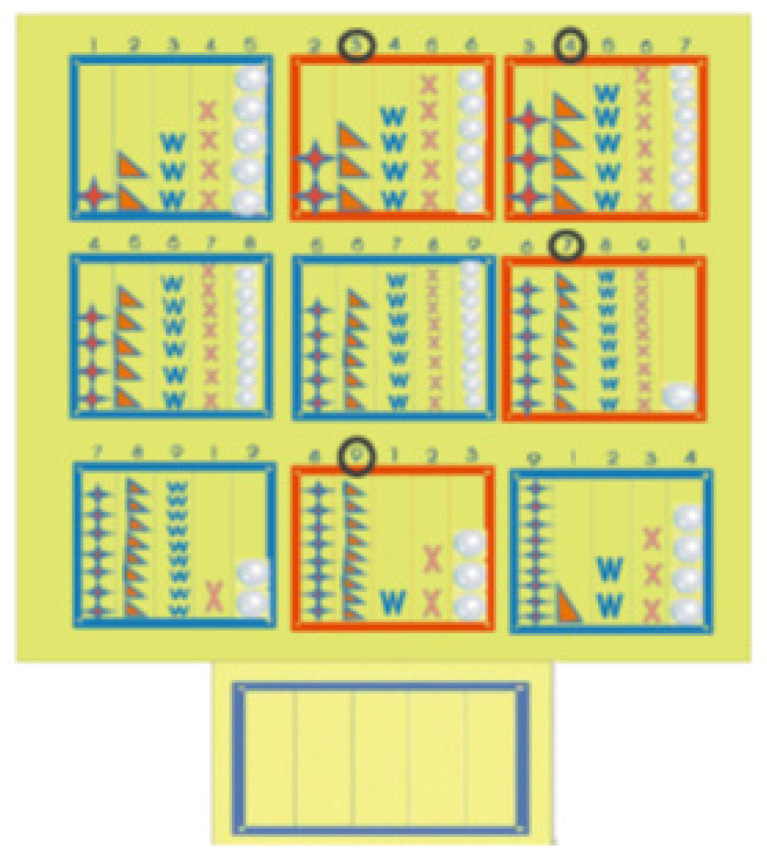
Graphical pad of login process of server voice authentication.

The use of extra equipment to strengthen the MPASs security and eliminate shoulder surfing attacks has increased. If the equipment is inexpensive and does not pose any difficulty to the users, it will be more acceptable and practical. Hassan et al. [163] proposed an MPAS that uses a headphone and a graphical password to stop any chance of a shoulder surfing attack. The user listens to the server voice telling a random number used in a formula to process the password, which is then entered graphically using a numeric type graphic pad as shown in Figure 13. This graphic pad has many symbols, which is why the user clicking on the screen to enter the password will never let the shoulder surfing attacker know what password was typed. Then, for the following authentication, the server voice changes the number, and the number pad also changes its numbering sequence along with the symbols and their orders. These are the proposed techniques addressing the issue of shoulder surfing attacks and duress attacks. This problem of shoulder surfing and duress attacks is an increasing one and poses threats day by day to users around the world while authenticating their precious property or assets.

Alhothaily et al. [30] proposed a technique combining the graphical password and a one-time password system. Users register with a unique username and draw a shape on a 4×4 pattern lock. In the login process, the user enters their username and draws a pattern; if the given data are correct, then a 4×4 grid of images appears, containing two user-pass Images and other distracting images to divert the attention of a bystander or a hacker. Codes associated with the correct images are automatically generated. The user has to recognize the correct images in their brain and does not have to click on the image but enter the code related to the correct pass image. In this way, the shoulder surfer does not know which images have been selected. This technique provides a good solution against shoulder surfing attacks common in public places.

Afzal et al. [164] proposed a technique where the user needs to process their PIN code whenever they log in to the system. The user has to process (mathematical operation) their PIN code digits with the server’s given numbers during every session.

The unique technique proposed in Imtiaz et al. [165] integrates machine learning and behavioral authentication, and prevents shoulder surfing, as the arithmetic operation is hidden by a hand on the screen. When a user places their palm on the screen to hide the code, the system displays the arithmetic operation and performs the calculation in their mind. The user is shown a public pattern, but the machine learns their touch dynamics and postures. The focus is on providing an additional layer of defense to save the users’ authentication processes. Table 16 summarizes the process authentication schemes.

#### 5.8.1. Opportunity

Process password authenticating schemes provide an extra layer of security. All authentication schemes discussed under this category require the user to log in with new credentials whenever they log in. This offers good protection against attacks such as guessing and shoulder surfing attacks. The user must process their password using the given server numbers. This processing should be simple enough not to require extra time to authenticate.

#### 5.8.2. Entropy in Security Usability Tradeoffs

Random passwords maximize the entropy by human bias, while processed authentication enhances user-generated credentials’ resilience. These authentication mechanisms continue to evolve by addressing embracing modern cryptographic techniques and usability challenges. Table 17 summarizes the comparison of processed authentication and random password authentication in terms of entropy and usability.

### 5.9. Augmented Reality Authentication

Augmented reality is a technique that eradicates attacks such as shoulder surfing. A device is used in which the user can only see what is on the screen or which keys are shown on the keyboard with different layouts. Nearby people, which can be potentially dangerous, cannot see what is displayed on the screen or the typed password [168]. As the number of augmented applications grows, one factor that has gone unnoticed is the privacy and protection of augmented systems. Users’ behavior in a virtual reality environment differs significantly from that for other digital devices such as smartphones or computers [169].

L’Yi et al. [170] proposed a desktop interface for writing subtly, i.e., to misdirect the observer’s attention from the authentic text. It is used to write personal messages in crowded places that misdirect the attackers from the real action.

**Figure 14:**
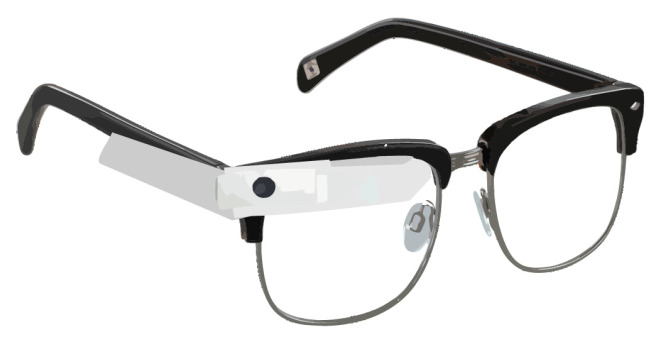
Design of Google Glass.

Seo et al. [171] proposed a system that uses a particular device, i.e., Google Glass (Figure 14), which deals with the concept of augmented reality and contains many properties, as it can interact with the user through voice, touch, and gestures. This technique is also beneficial when using an ATM or credit card in an open environment. The user enters their PIN using Google Glass, followed by the password, and then chooses the existing operation.

Zhang et al. [172] proposed a system based on authentication through an augmented reality display. The proposed system uses an augmented reality headset and a controlling device. The display is given through the headset, and the gesture device is responsible for recording the gestures and input. The user has to wear the device to input any gestures or data. After wearing it, the user has to execute numerous taps to record a pattern that will be a signature for that user and is called labeled sensor data. A model that corresponds to a particular user is created. Then, the user has to set a password, after which the headset will provide a virtual keyboard for the operator to enter the password. This virtual keyboard will have a numeric pad with eight keys from zero to seven, among which the user must set or insert the password. This proposed approach effectively prevents shoulder surfing attacks.

Olade et al. [173] paid central attention to the scheme of protection required to approve a user’s identity utilizing a variety of familiar characteristics that distinguish the user from other users in a virtual and augmented reality environment. Identifying the task comes first, followed by identifying the individual in the identification process. Machine learning was used to test 65,241 datasets regarding the movements of hands, head, and eyes to develop a continuous biometric authentication system, and it achieved an accuracy of 98.6%.

Corbett et al. [174] introduced GazePair, a new pairing system that enhances previous local pairing strategies with an efficient and user-friendly protocol. GazePair employs eye tracking and a spoken key sequence cue (KSC) to generate 64-bit symmetric, identical, and separately generated encryption keys. GazePair improves pairing success rates and timeframes compared to the current approaches. Additionally, it was also demonstrated that GazePair can support several users. Finally, GazePair can track eye gaze on any mixed-reality (MR) device.

The work Park et al. [175] aimed to create a non-contact authentication system employing ErPR epochs in an AR environment. Thirty participants were offered a quick visual presentation with familiar and unfamiliar human photos. ErPR was compared to Event-related Potential (ERP). ERP and ErPR amplitudes for familiar faces were substantially higher than those for strangers. The ERP-based authentication system achieved flawless accuracy using a linear support vector machine classifier. A quadratic discriminant analysis classifier trained on ErPR characteristics achieved 97% accuracy and had minimal false acceptance (0.03) and false rejection (0.03) rates. ERP and ErPR amplitudes had correlation values ranging from 0.452 to 0.829, and Bland–Altman graphs indicated good agreement. Table 18 summarizes the augmented authentication schemes.

#### 5.9.1. Opportunity

Augmented devices provide a stronger shield against attacks but are expensive [171]. Users need to carry an extra device for authentication, which is sometimes a problem. If the additional device is lost or stolen, then authentication using the schemes mentioned above in this section is impossible. So researchers should find a solution to get rid of the extra or augmented device, which should be small enough to carry.

#### 5.9.2. Entropy in Security Usability Tradeoffs

The entropy in augmented reality-based authentication is determined by interaction space, degrees of freedom, and temporal factors. Practical entropy can be low due to user behavior, device limitations and accessibility. Table 19 summarizes the comparison of augmented reality authentication in terms of entropy and usability.

### 5.10. Fingerprint Authentication

Since data security in mobile devices against hackers is a serious issue nowadays, the fingerprint is one of the unique human characteristic-based approaches that may provide better solutions in this regard [177]. Fingerprint recognition technology is being thoroughly studied and used for biometric authentication. A fingerprint is a unique pattern of slopes and valleys on a user’s fingertip used for authentication. Every individual, including identical twins, has a unique fingerprint. Capacitive fingerprint scanners have been used for identification in mobile devices, such as the iPhone and Samsung [6,178]. These scanners use capacitive proximity sensor matrices, with each finger ridge wider than the intervals between them.

Nguyen and Nguyen [179] suggested utilizing computer vision techniques in picture preparation to improve image quality and speed up classification in automatic fingerprint recognition systems with large datasets. However, this approach increases computation time. The combination of enhancements reduces the amount of comparisons in these systems. The Random Forest (RF) model outperformed SVM classifiers on the FVC 2000, 2002, and 2004 databases, with an accuracy of 96.75%.

Kumar and Priyanka [180] used Image Enhancement Techniques (IETs) to improve fingerprint photographs, resulting in more accurate feature extraction data. This review discussed the various IETs used in fingerprint recognition (FPR) systems. This article explained how enhancement strategies can increase the accuracy and reliability of feature extraction in fingerprint biometric systems.

Chen et al. [181] proposed an enhanced image quality classification approach that rejects erroneous input during preprocessing, reducing reaction time for fingerprint-on-display (FoD) applications. The approach was tested with a self-assembled dataset of 50,130 fingerprint pictures from FoD sensing and achieved 95.83% accuracy.

Sun et al. [182] investigated presentation attack detection (PAD) in fingerprint identification systems, highlighting the difficulty of separating legitimate users from fraudulent attempts in biometric systems. The authors provided a novel method using optical coherence tomography (OCT) features to detect attacks with a 4% Equal Error Rate (EER), similar to biometric systems prioritizing precise identification. Table 20 summarizes the fingerprint authentication schemes.

#### Entropy in Security Usability Tradeoffs

The entropy in fingerprint authentication systems indicates the difficulty of replicating or guessing the unique biometric feature. The entropy in fingerprint authentication depends on capture resolution, template creation, and fingerprint features. Table 21 summarizes the comparison of fingerprint authentication in terms of entropy and usability.

### 5.11. Face Recognition Authentication System

Biometric applications, including facial recognition, are increasingly vital in smart cities. Scientists and engineers worldwide are working to develop more precise algorithms and procedures for these systems and their practical applications. All security systems must secure all personal information. The most popular kind of recognition is the password. Biometrics are increasingly used for face recognition tasks due to technological advancements and security algorithms [183]. Face recognition is a popular study topic in computer vision and pattern recognition, with practical applications such as identification, access control, forensics, and human–computer interaction. Identifying a face in a crowd raises ethical concerns and problems about personal freedom. In recent years, numerous methodologies, algorithms, approaches, and datasets have been proposed for studying limited and unconstrained face recognition.

Li et al. [184] proposed sibling attack using a closely comparable task as the sibling task to generate strong adversarial attacks against face recognition tasks in a black-box context. Based on theoretical and quantitative studies, sibling attack chooses attribute recognition as the job. Sibling attack optimizes adversarial gradient information by constraining cross-task features to the same space, using a joint-task meta-optimization framework to improve gradient compatibility, and employing a cross-task gradient stabilization method to reduce oscillations during attacks. Extensive trials show that sibling attack surpasses state-of-the-art FR attack approaches by a significant margin, increasing ASR by 12.61% and 55.77% on average for pre-trained FR models and two popular commercial face recognition systems.

Dang [185] proposed the revised architecture utilizes depth-wise separable convolution to reduce model size and computational space while maintaining excellent accuracy and processing performance. Identifying individuals entering and exiting an area on mobile devices can be challenging due to limited memory and storage space. The technique achieves over 95% accuracy on a small dataset of original face photos, making it effective for practical applications. The obtained frame rate (25 FPS) is superior to neural network-based facial recognition methods.

Opanasenko et al. [186] presents a method for recognizing faces in mobile devices using an ensemble approach to pattern recognition, resulting in high accuracy. This technique breaks down the core algorithm into two operators: recognition and decision rules. The recognition operator estimates the test object’s proximity to the specified classes. The decision rule uses these estimations to assess if the tested item belongs to one of the specified classes. The recognition operators are organized as a linear polynomial. The polynomial parameters are determined by solving a multiparameter optimization problem. Open facial picture databases were used for experimental experiments.

Table 22 summarizes the face recognition authentication schemes.

#### Entropy in Security Usability Tradeoffs

The entropy in face recognition enables the system’s ability to identify between different users based on their facial features. Entropy depends upon algorithm complexity, facial features, and the resolution (quality) of the image capture device. Table 23 summarizes the comparison of face recognition authentication in terms of entropy and usability.

### 5.12. Anonymous Authentication Schemes

An application provider uses anonymous authentication schemes to offer services to its users several times after they have authenticated themselves anonymously. These privacy-preserving cryptographic algorithms rely on a secret key in a trusted platform module [187]. Some common techniques of anonymous authentications are zero-knowledge proofs and cryptographic algorithms. Zero-knowledge proof is a cryptographic methodology that protects privacy and data by safeguarding users’ identities and allowing them to utilize services anonymously. It has many applications, including authentication [188]. Cryptography is one method for ensuring the anonymity, authentication, credibility, availability, and authenticity of data users, and the data’s security and privacy. Cryptography is a cryptographic technique that provides the maximum security and secrecy of data conveyed via the communication channel by employing the same key for encryption and decryption [189].

Garg et al. [190] aims to enable users with limited computational resources to outsource evidence generation to untrusted servers while maintaining anonymity. The essential need is that these servers generate proofs faster than consumers. To achieve this goal, they developed zk-SNARKs-as-a-service (zkSaaS), a framework that accelerates zk-SNARK processing. Their technology distributes proof computation across numerous servers, resulting in shorter runtimes than a single prover. Furthermore, the prover’s witness’s privacy is protected by a minority of cooperating servers.

Baldimtsi et al. [191] created zkLogin, a mechanism that uses identity tokens from popular OpenID Connect sites like Google and Facebook to authenticate transactions. zkLogin’s signature mechanism relies solely on the signer’s existing OpenID credentials. This dramatically enhances the user experience because users no longer need to memorize a new password and can utilize their existing accounts. zkLogin offers excellent security and privacy guarantees. Unlike previous works, zkLogin’s security is purely based on the underlying platform’s authentication process, eliminating the need for additional trusted parties. As the name implies, zkLogin uses zero-knowledge proofs to hide the sensitive link between a user’s off-chain and on-chain identities, even from the platform.

Indushree et al. [192] proposed mobile chain, a secure blockchain-based authentication system for mobile contexts. The mobile chain is designed to safeguard user privacy and provide proven security features like authentication, anonymity, untraceability, confidentiality, data integrity, and decentralization. The security framework was implemented on the Ethereum blockchain platform, and smart contracts were written in Solidity programming language. Mobile chains outperform mobility networks in terms of security threats. The authentication framework has undergone formal security verification using Automated Validation of Internet Security Protocol and Application (AVISPA). The suggested approach retains performance gains, is computationally efficient, and may be implemented in resource-limited wireless and mobile contexts.

Chaudhary et al. [193] introduced a secure three-party post-quantum key setup mechanism for mobile devices. The proposed three-party key exchange protocol uses an authenticated shared key that can be renewed periodically to ensure forward secrecy. This protocol allows two parties to establish a shared session key even in the face of quantum adversaries, allowing for safe communication over unsecured networks. The protocol ensures anonymity as both sides communicate using masked dynamic identities.

#### Entropy in Security Usability Tradeoffs

The entropy in anonymous recognition enables the system’s ability to identify between different users based on their facial features. Entropy depends upon algorithm complexity, facial features, and the resolution (quality) of the image capture device. Table 24 summarizes the comparison of anonymous authentication in terms of entropy and usability. Table 25 summarizes the anonymous authentication schemes.

## 6. Datasets and Performance Evaluation

The lack of publicly available datasets hinders the methods used for smartphone continuous authentication. For example, a small group of participants was utilized in various datasets to record the behavioral data. The best authentication scores may always be obtained from small-scale datasets [194,195]. Furthermore, only a tiny number of datasets [196,197] collected biometric data from big member groups. These datasets were recorded in a laboratory or other controlled setting. Because of this issue, long-term authentication errors were common in the systems in place, as they required a broad user base, and researchers found it challenging to create a substantial public dataset in an uncontrolled setting.

A recent MIT study found that many popular AI datasets have labeling errors, highlighting the importance of addressing dataset quality issues [198]. The quality evaluation process established in [199] identified data and metadata quality issues. Data quality concerns include redundant, inconsistent, missing, and erroneous data. Metadata quality issues include incomplete and imprecise metadata for metrics or entities. An assessment scale is created based on the problem definition to conduct quality assessments. The significance of datasets in machine learning research cannot be emphasized. Datasets can hinder algorithmic development and scientific progress. However, some benchmark datasets, such as ImageNet for visual object recognition and GLUE for English textual understanding, have led to significant advancements in the field.

Additionally, many people could be reluctant to submit their biometrics due to privacy and security concerns. Thus, this allows researchers to produce benchmark datasets that are free of charge and available to the general public. Adopting suitable characteristics for authentication on a smartphone is difficult since choosing redundant elements can impact the performance of authentication as a whole. The goal is to enhance user authentication performance by creating a suitable similarity-matching method for authentication. MPAS, especially the behavioral authentication schemes, depends on the datasets’ availability; some popular datasets are briefly described below.

### 6.1. MNIST Handwritten Database

A training set of 60,000 examples in the MNIST handwritten digits database, accessible from this list, and a test set of 10,000 examples. The digits have been normalized and centered in size in a fixed-size picture. This database has been widely used by researchers [200,201] for handwritten digit authentication schemes. The MNIST database was created from the original NIST database, hence the name modified NIST or MNIST. The dataset includes 60,000 training photos (some for cross-validation) and 10,000 test images from the same distribution. All these black and white digits are size normalized and centered in a fixed-size image with the intensity’s center of gravity in the middle. Thus, the dimensions of each image sample vector is 28 × 28 = 784, with each member being binary. This small database allows users to experiment with machine learning and pattern recognition on real-world data with minimal preprocessing and formatting.

### 6.2. e-BioDigitDB

For 93 users, the e-BioDigit database contains online handwritten numerical digits from 0 to 9 obtained using a Samsung Galaxy Note 10.1 general-purpose tablet. All samples are collected using the touch of a finger as input, so only information relevant to X and Y spatial coordinates is considered. Researchers in Tolosana et al. [202,203] used this dataset in their experiments to verify and validate their work.

### 6.3. MobileTouchDB

MobileTouchDB is a modern mobile touch biometric database of handwritten characters that exceeds 64K online personality trials produced by 217 users [204]. The database examines an unsupervised mobile schema with no position, posture, and device limitations.

Evaluating any authentication system depends on three main factors, i.e., security, usability, and privacy.

### 6.4. Security

The rapid rise of the internet and mobile applications has increased user requirements. Users desire user-friendly authentication and reliable system security. Users expect a convenient and secure authentication system. An authentication system should be simple enough for everyone to use. A defense mechanism improves system robustness but reduces usability. As discussed in Section 3, authentication systems risk different attacks, mainly shoulder attacks, which do not require the hacker to gain any extra knowledge of software, etc. Therefore, any MPAS should be able to resist any attack.

### 6.5. Usability

Evaluation of MPAS with the following requirements is essential for usability:

#### 6.5.1. Admissibility

Users should widely acknowledge the designed biometric authentication system, including acceptance of the collection scheme (in the case of behavioral data). Evaluating an authentication system’s performance requires considering user preferences. User preference refers to their willingness to accept system UI design and performance, among other factors. The result is a combination of user cognition and psychological feelings.

#### 6.5.2. Extra Tool

If a user carries any extra device for authentication, it should not burden them. Additional special equipment is needed for the collection of biometric data. The availability of each component might influence the level of usability and acceptance among different types of users. EEG sensors are expensive and require a stationary and tranquil environment for use. However, significant advancements in recent years have made mobile cameras and webcams more widely accepted.

#### 6.5.3. Easy Retrieval

Easy retrieval is calculated by the mental effort needed to retrieve and deduce the authentication key that the user would use. The MPAS should be easy enough for the user to recall in every session.

### 6.6. Privacy

The MPAS should be secure enough not to leak users’ login credentials at any stage. Two main loopholes can usually cause login information leakage: when a user is logged-in in a public environment, or stealing in a network environment during storage or transmission. Hackers can exploit an unprotected network to gain access to multiple services, resulting in a total invasion of privacy.

## 7. Challenges and Future Directions

This section describes various challenges with the existing MPAS and future directions as a possible remedy to the problems in the existing mechanisms.

### 7.1. Challenges

Now, authentication is as essential as it was in the past. In this modern era, users will mostly focus on biometrics to supplement traditional passwords in device protection and authorization matters. An authentication system should promise modern users security and ease of use when accessing sensitive data. A combination of various authentication schemes provides excessive security when verifying the user. In this paper, the most user-friendly authentication schemes mentioned involve minimal user interaction, such as the strategies requiring minimal user effort. As the tradeoff between usability and security, designing a fallback MPAS that is both memorable and powerful is challenging work [205]. Unfortunately, MPAS research often focuses on poor adversary frameworks, resulting in excessively optimistic security performance [206]. To alleviate the discomfort, attention must be focused on schemes that are more effective, difficult to compromise, and most importantly, simple to use so that users can concentrate on the activities that take place behind the authentication interface rather than the visible authentication interface [207].

Based on this review, several open authentication research problems that should be investigated have been identified. The evolution of a modern mobile device’s computing capabilities and storage size has transformed it into a multi-purpose smart gadget for personal and business use. The increased use of this gadget necessitates implementing a secure and efficient authentication method. Password, PIN, and swipe patterns are standard user authentication methods for mobile devices. Entry-point face and fingerprint recognition have also gained popularity in recent years. However, these authentication techniques cannot authenticate a user after the first login session. This limitation may expose the device to information theft and leakage if an unauthorized user can bypass the initial login session.

#### 7.1.1. Usability

Usability is essential to secure MPAS because it minimizes resistance and promotes acceptance. The review shows that traditional passwords, pattern-unlocked schemes, and augmented reality authentications are helpful because they ease the perception. Users of the systems studied tended to accept them, although there may be adverse effects on the types of users in the system. For instance, carrying multiple tokens has increased perceived personal accountability for effective authentication, making the danger and discomfort related to disappearance and theft significant for the candidates [19]. A poor MPAS design cannot promote simple deployment, quick integration of different components, and versatile reconfiguration. Therefore, the usability studies have been limited. There is no qualified analysis of various authentication models, e.g., diverse users in terms of numbers and types, or modifying the selection procedure. Therefore, it should be a priority to address the design problems, as it will provide the basis for further analysis of usability and security issues. Managing privacy, system efficiency, and usability remains an essential and open topic. It is crucial to address how to detect user behavior and prevent attacks such as shoulder surfing, replying, and spoofing since they are straightforward to launch.

#### 7.1.2. Security

In a real-life world, the disclosure of privacy and related security is still an open issue. Privacy disclosure in network transmission can be resolved by improving data’s unlinkability, non-invertibility, and revocability. In addressing the exposure of confidential data in real life, we must educate users about privacy. Realizing authentication mechanisms that react to environmental changes creates new vulnerabilities and attack avenues. Device theft is an issue when the user carries any external authentication device [208]. Data privacy is a severe issue for behavioral authentication, especially if such information is not stored locally or if algorithms are outsourced to third parties. Attacks aimed at system components based, for example, on machine learning techniques compel an illegitimate user to be incorrectly classified as authentic.

### 7.2. Future Direction

To concentrate on implementing a functional and reliable MPAS to preserve privacy, this article proposes a range of potential research directions.

There is a need for research on a safe biometric MPAS. To achieve high-level user acceptance and broad adoption, usability improvement and reliability insurance are worth special exploration. The commonly used biometric authentication system based on static features, such as touch ID, probably needs to include a means of detecting liveliness. An important research topic worth studying is protecting private user biometric information, especially when biometric specifications are saved in a foreign entity that can not be fully trusted. The usability of any MPAS could be influenced by various factors, including user-device interaction design, method of data collection (in the case of biometric MPAS), and protocol design of authentication. Source-limited mobile device authentication costs should be taken into account. Most mobile devices have restricted electricity resources, computing power, and storage room.

Authentication protocols can be built with cryptography that is not vulnerable to phishing, but the challenge is to pack them up efficiently to be easy to use. There is a need to find alternatives that do not require users to be security professionals. However, to have guidelines in some way to help them along is a positive thing.

Multi-factor authentication (MFA) signifies integrating at least two different authentication factors. One emblematic factor is what you are (PINs and passwords); other factors can be the user’s choice. Adding a secondary factor to the primary factor strengthens the user’s privacy, as the traditional single-factor authentication opens up an imposing violation routine that can be easily compromised. With MFA, multi-layers of protection can be achieved. Unfortunately, ignorance is shown whenever there is a question of managing the passwords and authentication scheme. At present, the users adopt a single authentication factor, but there is an absolute need to switch to MFA. The primary obstruction in implementing MFA is the delusion about the required external hardware or other hardware tools. The correction of this delusion may result in outstanding authentication schemes.

Owing to the many authentication methods covered in Section 3, it is evident that most authentication factors lack independent reliability, are susceptible to various attacks, or have an impact on output due to fault tolerance. Because the majority of authentication elements on their own are weak, password-based authentication is still widely used. Another common factor must be added to their procedures to fortify them against additional attacks and protect them if the first step is bypassed. Multi-factor authentication is a series of actions that strengthen the user login process. However, increasing the system’s complexity will impact other areas, such as performance and usability. Password rules are notoriously abused by users who must remember various changeable passwords. Although a token alleviates the problem of forgetting passwords, the user must remember to carry the physical item, which might be difficult sometimes. Although biometrics eliminate the problem of forgetting, incorrect non-match findings and low battery life may cause difficulty for some users. Because the application determines the tolerable cost of an authentication system, the user should justify the cost of an attack on their private data. Depending on how the user rates their data in terms of security levels, the installation of security to lower the danger of a successful assault must be determined accordingly. If the data are not as critical, weaker security methods such as passwords/PINs may be sufficient, owing to the concept that poor security is always preferable to no security. To allow application-specific access control decisions, smart authentication systems will, in the future, rely on many factors or at least a combination of two authentication approaches.

Based on earlier research and analysis, we offer the following four-step procedure for presenting an efficient authentication scheme for smart mobile devices.

- Specify the authentication mechanisms that can be utilized.
- Identifying system vulnerabilities and possible interconnections.
- Security analysis techniques are used to validate the efficiency of the given approach.
- Analyzing performance such as user and server computational costs.

#### 7.2.1. Development and Implementation of an Efficient Mechanism for Balancing Security and Privacy

Most secure authentication systems are based on biometrics such as iris, fingerprints, and voice, or traditional authentication credentials like passwords. The investigation reveals that if the user ID is hacked, authentication scheme mechanisms become worthless [209]. Balancing security and privacy when implementing remote authentication techniques is critical in a contemporary global structure. It is necessary to establish an acceptable security and privacy tradeoff limit. Without adequate protection, there can be no privacy implementation [210].

#### 7.2.2. Development and Implementation of an Energy-Efficient Authentication Mechanism for Diverse Applications

Cloud computing has now given way to the Internet of Things domain, where an application may request that the user’s every move be tracked to aid in remote applications such as healthcare. These information types are private to the individual and must be secured from security breaches [211]. Sensors for detecting and data collectors for relaying information consume very little energy. Hence, authentication techniques utilizing them must be highly energy efficient [212].

#### 7.2.3. Development and Implementation of Cost-Effective Biometrics Mechanism for Recording Features

The equipment to record the features must be designed to be energy efficient and accurate [213]. As a result, using any specialist gadget is frequently regarded as an additional cost. Only increased efficiency in biometrics feature extraction can justify this extra cost [214].

#### 7.2.4. Development and Implementation of Reliable Authentication Mechanism

Reliability depends upon the implementation of factors involved in the authentication process and the number of factors used. Reliability can be achieved by reducing the error rate and improving the robustness [215]. Reliability is directly linked with the privacy and security of an MPAS. So, by strengthening the privacy and security of an MPAS, the scheme becomes more reliable.

## 8. Conclusions

The MPASs mentioned in this article are the easiest to use and require the least user interaction. Using random characters for the password is a realistic way to make text-based authentication easier, but using such complex passwords is also off-putting to a mobile device user. The four obstacles to an effective MPAS are the cost of the authentication system, the user’s ease during the authentication, the protection provided by the authentication system, and the privacy provided by the authentication system. Consequently, the MPAS combines all these to the best possible dimensions and is a better system. Table 26 shows the more popular authentication schemes; the least expensive forms of authentication are password/pin and pattern-based authentication, which provide medium security at a low cost. However, these authentication schemes are less secure because users tend to recall their hidden codes using simple passwords. Biometric MPAS provides maximum privacy since users do not need to worry about anyone peaking when they enter their credentials. This paper presents various existing password attack-resistant techniques. Much effort has been made to make sure that the MPAS presented will be useful for researchers in this area now and in the future.

## Figures and Tables

**Table 1:** An overview of attacks on mobile devices.

Type of Attack	Reference
Shoulder attack	Eiband et al. [29]
Brute Force Attacks	Fujita and Hirakawa [51]
Dictionary Attack	Bosnjak et al. [35]
Replay Attacks	Syverson [37]
Phishing Attacks	Uusitalo et al. [40]
Key loggers	Raza et al. [43]
Guessing Attacks	Biddle et al. [44]
Smudge Attacks	Patel and Patel [52]
Electroencephalography Signals	Kumar et al. [49]

**Table 2:** A summary of recent relevant survey papers in the field.

Ref.	Authentication Schemes Discussed	Limitations
Ali et al. [25]	This evaluation research shows that the 2FA in mobile devices	The review only address one time of authentication
Mayrhofer and Sigg [53]	The suggested classification of adversaries provides a powerful and practical adversary model that enables a comparable and precise classification of security properties in mobile phone authentication	Focuses on adversary models only
Alotaibi et al. [26]	This study highlights the need to look into the sensitivity level of the application and determine whether or not a particular application needs to be protected when deciding when to authenticate the mobile user	The review focuses only on touch authentication mechanisms
Aslam et al. [54]	The suggested authentication procedures are reviewed in this work, along with their advantages and disadvantages in terms of computing cost, guaranteed security, and privacy features	Hybrid authentication technique for pertinent authentication is missing
Faruki et al. [57]	By providing an understanding of the advantages and disadvantages of established research methodology, this review enables researchers to propose approaches for malware analysis, harmful app identification, and next-generation Android security	Focuses only on Android malware threats
Meng et al. [58]	The authors evaluate the current initiatives in the field of biometric phone authentication and assess how feasible it would be to implement them on touch-enabled smartphones	Biometric authentication techniques are discussed
Teh et al. [59]	This work aims to give some insights and a comparative analysis of the state-of-the-art in the field, covering experimental settings and evaluations, feature data representations, data gathering methodologies, and decision-making strategies	Touch biometrics on mobile devices are addressed
Sharma and Mishra [60]	This study explores the security of significant Android password managers, exposing flaws such as weak password generation, unprotected metadata, and phishing concerns. It makes recommendations to improve both user experience and security, as well as identifies future study areas	The brief oversimplifies critical security issues and provides insufficient context on individual vulnerabilities and suggested remedies

**Table 3:** Comparison of PIN and password authentication schemes in terms of entropy and usability.

Feature	PIN	Password
**Entropy**	Low (13–20 bits)	Higher (20–78+ bits)
**Usability**	Easy to remember and input	Harder to remember
**Vulnerabilities**	Shoulder surfing, brute force,	Phishing, Guessing, reuse
**Enhancements**	Randomized keypads	Strength policies

**Table 4:** Summary of behavioral authentication schemes.

Algorithm	Year	Findings	Limitations
SVM [82]	2013	The suggested technique consumes less energy while presenting quick and precise authentication.	Neglects context features Bello et al. [91].
KNN [83]	2017	KNN has good efficiency than SVM in all cases.	Analyzed small number of gestures [91].
CNN [85]	2018	ConvNet has the highest predictive accuracy in the tap behavior of the user than the algorithms compared.	Needs many layers to reach features layer. [91]
CNN [86]	2018	Three modalities for efficient identification and smart spoof recognition are used in the hybrid scheme.	User needs to carry an extra device for authentication.
DTW [66]	2017	DTW is used to authenticate smartphone users using PIN-writing behaviors.	The proposed scheme is restricted to few subjects and does not include any form of assessment.
LSTM RNNs [88]	2022	A comparative analysis was conducted on behavioral traits acquired through common mobile interactions like typing and tapping, alongside sensor data from various mobile sensors.	Although the study focuses on various background sensors, it may not completely investigate the possibilities of all available sensors on mobile devices. This shortcoming may limit the efficacy of the multimodal method.
LSTM [89]	2023	ABehavePassDB is a comprehensive mobile behavioral biometrics database that allows continuous authentication using background sensors and touchscreen interactions.	While BehavePassDB makes a significant contribution, the document does not address potential limits in the diversity of the user population or the types of devices used, which could affect the generalizability of the results.
LSTM [90]	2024	An innovative Continuous Verification System (CVS) adapts to user interactions in real-time, improving security and user experience.	The data collection is based on specific activities that may not capture the whole spectrum of user interactions with mobile devices, affecting the system’s capacity to generalize to different situations.

**Table 5:** Comparison of behavioral authentication schemes in terms of entropy and usability.

Feature	Behavioral Authentication	Traditional Authentication
**Entropy**	Moderate to High (20–50 bits)	Low to Moderate (13–52 bits)
**Usability**	High (Passive Monitoring)	Moderate (User Input Required)
**Vulnerabilities**	Noise Variability	Brute Force
**Enhancements**	Feature Diversification	Strength policies

**Table 6:** Summary of keystroke authentication schemes.

Ref.	Year	Findings	Limitations
Khan et al. [95]	2020	Challenge by developing an augmented reality-based app that resides on the attacker’s smartphone	Cannot resist mimicry attacks [101]
Zahid et al. [99]	2009	A user identification system that detects a cell phone user’s keystroke dynamics to identify authorized users from impostors	Degradation of response time to the mobile device that might affect user acceptance [102]
Buchoux and Clarke [97]	2008	Low computational requirements that can be used on a real computer have been shown by statistical classifiers, with faster response in both template generation and sample verification	only practical in scenarios with sufficient keystroke activity [103]
Saevanee and Bhattarakosol [98]	2009	Learn the potential of person behavioral biometrics, including finger pressure, hold-time and inter-key	Small number of participants were used for experimental study [102]
seob Hwang et al. [100]	2003	Keystroke dynamics-based authentication (KDA) provides better security against different attacks	Biometric measure lacks universality, uniqueness, permanence, precision, and acceptance because it simply communicates keyboard timing features [104]

**Table 7:** Comparison of keystroke authentication schemes in terms of entropy and usability.

Feature	Keystroke Authentication	Traditional Authentication
**Entropy**	Moderate to High (30–66 bits)	Low to Moderate (13–52 bits)
**Usability**	High (Passive Monitoring)	Moderate
**Vulnerabilities**	Replay, Mimicry, Noise	Brute Force
**Context Sensitivity**	High (Environment Dependent)	Low (Independent)

**Table 8:** Summary of touchscreen authentication schemes.

Ref.	Year	Findings	Limitations
Colley et al. [106]	2016	New changes to the existing pattern lock mechanism including the reuse of a node after it has already been used	Research is constrained by the small sample size and laboratory environment [113]
Eiband et al. [107]	2016	Replaces the traditional font of text messages with user handwriting	Not using inferential statistics [114]
Krombholz et al. [108]	2017	Improves digit PINs security by assigning binary pressure to digits in the series	Obtaining accurate force touch information can be challenging [115]
Chakraborty et al. [109]	2016	The proposed scheme works in a partly measurable setting where an auxiliary device is required for authentication	External instruments such as headphones are used to transmit the challenge value [116]
Vaddepalli et al. [110]	2020	Proposed PassO, an interface regarding touchscreen devices depending on the circular grid	Comprehensive usability evaluation is missing that should include more users to judge the proposed scheme
Gattulli et al. [111]	2023	Proposed a feature extraction method that includes the Signal Vector Magnitude and evaluates various machine learning models, including 1-class and 2-class SVMs	The conclusions drawn from the study may not be generalizable due to the limited number of users in the dataset
Finnegan et al. [112]	2024	The findings indicate that touch gestures and movement are the most commonly used biometric methods, with a significant reliance on accelerometer and touch data streams	The review is limited by the low quality of the included studies, which hampers the ability to draw robust conclusions about the efficacy of behavioral biometrics

**Table 9:** Comparison of touchscreen authentication schemes in terms of entropy and usability.

Feature	Touchscreen Authentication	Traditional Authentication
**Entropy**	Moderate to High (25–50 bits)	Low to Moderate (13–52 bits)
**Usability**	High (Natural Gestures)	Moderate
**Vulnerabilities**	Shoulder Surfing, Smudge Attacks	Brute Force
**Context Sensitivity**	High (Environment Dependent)	Low (Independent)

**Table 10:** Gaze-based authentication schemes.

Ref.	Year	Findings	Limitations
Katsini et al. [122]	2018	Two-step process for analyzing the complexity of graphical passwords generated by users based on the behavior of the eye-gaze during the creation of passwords.	Small sample size for the recall-based scheme.
Khamis et al. [121]	2016	Allows user to create passwords with multiple switches between input modalities	Thermal attacks on this technique would be ineffective in revealing the PIN [127].
Abdrabou et al. [123]	2019	A good balance between usability and protection is provided by gaze.	Multimodal authentication, which has the disadvantage of making password entering more difficult. It may have an impact on the password symbols’ memorability [128].
Abe and Yamada [124]	2020	Quality assessment scheme on eye movement authentication.	Continuous lines of sight, such as the trajectory prediction estimation in reading the text, should be evaluated and investigated.
Constantinides et al. [125]	2020	Using eye gaze changeability and concentration on hotspots throughout graphical password generation, calculate the intensity of user-chosen graphical passwords.	Gaze-based security solutions require highly accurate gaze estimates to be completely implicit and work without the user’s participation.
Namnakani et al. [126]	2023	They discussed GazeCast’s potential applications in a range of public venues, as well as the importance of further study to improve its usability.	The study’s findings may be limited by the small sample size and the lack of exploration into varying environmental factors that could affect gaze tracking accuracy.

**Table 11:** Comparison of gaze-based authentication schemes in terms of entropy and usability.

Feature	Gaze-Based Authentication	Traditional Authentication
**Entropy**	Moderate to High (40–66 bits)	Low to Moderate (13–52 bits)
**Usability**	Moderate (Hands free)	Moderate
**Vulnerabilities**	Replay	Brute Force
**Context Sensitivity**	High (Environment factor)	Low (Independent)

**Table 12:** Graphical authentication schemes.

Ref.	Year	Findings	Limitations
Wiedenbeck et al. [144]	2005	Graphical passwords provide more security against different attacks	There are no artificial predefined borders around portions of the image that the user can click within Wiedenbeck et al. [141]
Cain and Still [148]	2016	Degraded images are used for authentication that decreases the login time to 14 s	The proposed study uses a limited number of users for user study
Wiedenbeck et al. [149]	2006	The framework helps a user with the ease of verifying the graphical password in an endangered environment as the user indirectly selects the images for their password	An additional icon should be created to make the security settings more realistic
Yu et al. [150]	2016	3D graphical password for mobile authentication	Shoulder surfing attacks are successful against 3D passwords Izadeen and Ameen [153]
Irfan et al. [151]	2018	Combination of text and graphical password	Waiting time for the synchronization of images results in lengthy login time Das et al. [154]
Khan et al. [152]	2023	The proposed scheme incorporates multiple factors, including simple arithmetic operations, machine learning for hand gesture recognition, and medical images for recall, to create a user-friendly and memorable authentication process	The study’s findings may be limited by the small and homogeneous sample size, which could affect the generalization of the results

**Table 13:** Comparison of graphical passwords authentication in terms of entropy and usability.

Feature	Graphical Authentication	Traditional Authentication
**Entropy**	Moderate to High (25–40 bits)	Low to Moderate (13–52 bits)
**Usability**	High (Visual memory)	Moderate
**Vulnerabilities**	Smudge, shoulder surfing	Brute Force
**Context Sensitivity**	High (Environment Dependent)	Low (Independent)

**Table 14:** Color-based authentication schemes.

Ref.	Year	Findings	Limitations
Potey et al. [156]	2016	Authentication using color code that gives two-step authentication for use	Directed towards shoulder surfing attack if the attacker records the authentication process or observes the authentication process a few times The authentication process takes time because of its many stages
Jain et al. [157]	2017	combination of Pass faces, Pass points, and Story for strong authentication	Color combination confuses the user to get authenticated
Chiang and Chiasson [158]	2013	Addressed input accuracy problems without the need to remember images while preserving a password space that is sufficiently protected	The new version is unlikely to be identical to the original; redrawing a picture is harder than retyping words Fong and Poet [162]
Gugenheimer et al. [159]	2015	ColorSnakes can be used to add extra protection to specific applications like images, emails, and banking	The shift from a standard four-digit PIN to a four-digit + color or five-digit + color PIN may have affected memorability
Woods and Silvennoinen [160]	2023	Allowing users to select colors instead of pre-selected ones encourages them to build more personal and meaningful passwords. Color can improve password security by increasing entropy.	It excludes colorblind participants from the study, limiting the generalizability of the findings regarding color-based password memorability and security for all users
Selamat et al. [161]	2024	Color-based authentication is less vulnerable to visual hacking and predicting passwords due to its complexity and lack of link to user information than text-based identity verification, which usually contains personal information in the password	it may reduce user friendliness, particularly for colorblind users, as it requires color selection for password input, complicating the authentication process

**Table 15:** Comparison of color-based authentication in terms of entropy and usability.

Feature	Color-Based Authentication	Traditional Authentication
**Entropy**	Moderate to High (5–10 bits)	Low to Moderate (13–52 bits)
**Usability**	High (Visual and Intuitive)	Moderate
**Vulnerabilities**	Replay, shoulder surfing	Brute Force
**Context Sensitivity**	High (Color Perception)	Low (Independent)

**Table 16:** Process authentication schemes.

Ref.	Year	Findings	Limitations
Afzal et al. [164]	2010	Change PIN code on every login session prevent shoulder surfing	Authentication process requires mathematical operation which is time-consuming
Prabhu and Shah [131]	2015	Text along with images are combined to create session passwords	The system can easily be compromised by taking a screenshot Alajmi et al. [69]
Hassan et al. [163]	2015	Combination of something user process authentication factor and graphical password	The resistance of SVGA to observation attacks is very weak Dan and Ku [166]
Alhothaily et al. [30]	2017	Using cryptography primitives, i.e., digital signature, hashing, and encryption, creating a secure authentication mechanism	The proposed approach is resistant to phishing attacks, replay attacks, and shoulder surfing attacks; nonetheless, key capturing poses a risk to the system [167]
Imtiaz et al. [165]	2022	The user is presented with a public pattern, but the system learns their touch dynamics and postures, including lying posture. The focus is on adding a layer of defense to secure users’ authentication processes.	A vital drawback of the article is that it relies on a limited sample size and participant diversity, which may affect the generalizability and robustness of the proposed behavioral authentication system across different user demographics and real-world scenarios.

**Table 17:** Comparison of processed authentication and random password in terms of entropy and usability.

Feature	Processed Authentication	Random Passwords
**Entropy**	Matches original input entropy	Low to Moderate (13–52 bits)
**Usability**	High (Minimal User Involvement)	Low (Difficult to Remember)
**Vulnerabilities**	Poor Salting, Hash Reversals	User Rejection
**Enhancements**	Cryptographic Algorithms	Password Managers

**Table 18:** Augmented authentication schemes.

Ref.	Year	Findings	Limitations
L’Yi et al. [170]	2016	A new desktop application that enables subtle writing	The keystrokes observed by the adversary through shoulder surfing individually or through the camera will easily get to know about the password written by the keys pressed
Seo et al. [171]	2017	Masked password that only includes offset numbers for the input password to be corrected	Google Glass device is not accessible to every user
Zhang et al. [172]	2017	Augmented reality gesture authentication, that is based on the interpretation that only the user is seen in augmented reality	User needs to carry extra device for authentication
Olade et al. [173]	2020	Biometric identification system in a virtual environment	There is a noticeable drop in the accuracy when considering cross-system behavior-based biometric authentication [176]
Corbett et al. [174]	2023	GazePair improves pairing rates and times compared to current approaches. In addition, we demonstrate that GazePair can support several users. GazePair is compatible with any Mixed Reality (MR) device that has eye gaze tracking.	GazePair’s reliance on a spoken key sequence cue (KSC) for out-of-band communication may create security concerns in public settings, as the low entropy of KSC (only 9 bits) could be vulnerable to eavesdropping if not adequately safeguarded
Park et al. [175]	2024	The ERP-based authentication system achieved 100% accuracy using a linear support vector machine classifier. A quadratic discriminant analysis classifier trained on ErPR characteristics had high accuracy (97%) and low false acceptance (0.03) and false rejection (0.03) rates. ERP and ErPR amplitudes had correlation values ranging from 0.452 to 0.829, and Bland–Altman graphs indicated strong agreement between them.	It does not address the potential security risks associated with using color-based passwords, particularly for users with color vision deficiencies

**Table 19:** Comparison of augmented reality authentication in terms of entropy and usability.

Feature	Augmented Reality Authentication	Random Passwords
**Entropy**	Moderate to High (10–22 bits)	Low to Moderate (13–52 bits)
**Usability**	High (Immersive and Intuitive)	Low (Difficult to Remember)
**Vulnerabilities**	Replay, shoulder surfing attacks	Brute force
**Enhancements**	Multimodal Inputs	Password Managers

**Table 20:** Fingerprint authentication schemes.

Ref.	Year	Findings	Limitations
Nguyen and Nguyen [179]	2019	This paper proposes a fingerprint classification approach that uses Random Forest and support vector machine machine learning methods. Both approaches use machine learning to classify items with high accuracy 96%.	The proposed system depends on the quality of the image, which might decrease its performance as the fingerprints are often noisy in real real-world environment
Kumar and Priyanka [180]	2019	Authors proposed different techniques to extract perfect quality of image for fingerprint authentication	There is limited discussion of computational efficiency, which is crucial for practical deployment in large-scale systems
Chen et al. [181]	2021	The proposed method using a self-collected fingerprint sensing picture dataset of 50,130 fingerprint images and discovered that it can achieve 95.83% accuracy, which is extremely useful for improving the system’s user experience	The complexity of computation in real-time is not addressed
Sun et al. [182]	2023	The suggested technique has a 4% Equal Error Rate (EER), greatly outperforming prior presentation attack detection methods	Spoof materials mimicking internal fingerprint structures challenges are not addressed

**Table 21:** Comparison of fingerprint authentication in terms of entropy and usability.

Feature	Fingerprint Authentication	Random Passwords
**Entropy**	Moderate to High (10–22 bits)	Low to Moderate (13–52 bits)
**Usability**	High (Immersive and Intuitive)	Low (Difficult to Remember)
**Vulnerabilities**	Replay, shoulder surfing attacks	Brute force
**Enhancements**	Multimodal Inputs	Password Managers

**Table 22:** Face recognition authentication schemes.

Ref.	Year	Findings	Limitations
Li et al. [184]	2023	The suggested sibling attack uses a closely comparable task as the sibling task to create highly transferable adversarial attacks against FR tasks in a black-box context	It raises ethical questions regarding the use of adversarial tactics to breach biometric security
Dang [185]	2023	The research proposes an enhanced FaceNet model with a MobileNetV2 backbone and SSD component	Under lighting and shadow conditions, the system accuracy can be reduced
Opanasenko et al. [186]	2024	Using an ensemble of recognition operators improved object recognition accuracy in the control sample compared to the best basic recognition method	The collective approach may take large computer resources, leading to slower processing times and decreased efficiency in real-time applications

**Table 23:** Comparison of face recognition authentication in terms of entropy and usability.

Feature	Face Recognition Authentication	Fingerprint Authentication	Random Passwords
**Entropy**	Moderate (15–25 bits)	Moderate to High (10–22 bits)	Low to Moderate (13–52 bits)
**Usability**	High (Contactless and Fast)	High (Immersive and Intuitive)	Low (Difficult to Remember)
**Vulnerabilities**	Spoofing, Deepfakes, Replay Attacks	Replay, shoulder surfing attacks	Brute force
**Enhancements**	3D Scanning, Liveness Detection	Multimodal Inputs	Password Managers

**Table 24:** Comparison of anonymous authentication in terms of entropy and usability.

Feature	Anonymous Authentication	Biometric Authentication	Random Passwords
**Entropy**	High (128–256 bits)	Moderate (10–30 bits)	Low to Moderate (13–52 bits)
**Usability**	Moderate (Requires Cryptographic Tokens)	High (Immersive and Intuitive)	Low (Difficult to Remember)
**Vulnerabilities**	Replay, Metadata Leaks	Spoofing, Template Theft	Brute force
**Enhancements**	Blockchain	Liveness Detection	Password Managers

**Table 25:** Anonymous authentication schemes.

Ref.	Year	Findings	Limitations
Garg et al. [190]	2023	This framework allows us to create bespoke protocols that improve the runtime of popular zk-SNARKs.	Distributing randomness multiple servers are required to run the said framework, which can be resource intensive
Indushree et al. [192]	2023	Mobile chain’s security research shows that it is resistant to security threats that mobility networks face	Significant computational resources and energy can be required due to the decentralized architecture of blockchain
Baldimtsi et al. [191]	2024	zkLogin provides other applications outside blockchains. It enables billions of individuals to create verifiable digital material, such as email addresses, using their current digital identities.	The proposed system can be exposed to potential risk, as zkLogin relies heavily on centralized Web2 identity providers like Google or Facebook for authentication.
Chaudhary et al. [193]	2024	The proposed protocol utilizes quantum-resistant cryptographic primitives, particularly those based on lattice structures, to ensure the forward secrecy and integrity of key agreement procedures in a three-party protocol	Managing keys securely in a three-party system across diverse mobile networks can be complex and may not scale well in real-world applications

**Table 26:** Authentication schemes performance.

MPAS	Security	Relative Cost	References
Password/Pin	Minimum	Less	Van Nguyen et al. [66], Ku et al. [67], Harbach et al. [216]
Behavioral	Maximum	Less	Li et al. [71], Frank et al. [72], Song et al. [83]
Keystroke	Medium	Less	Sun and Upadhyaya [93], Kambourakis et al. [94], Saevanee and Bhattarakosol [98]
Biometric Fusion	Maximum	High	Yang and Zhang [217], Zhang et al. [218], Gupta and Gupta [219]
Touchscreen	Minimum	Less	Eiband et al. [107], Krombholz et al. [108], Chakraborty et al. [109]
Gaze base	Maximum	High	Rajanna et al. [120], Khamis et al. [121], Abdrabou et al. [123]
Graphical Password	Maximum	Less	Sun et al. [143], Wiedenbeck et al. [144], Cain and Still [148]
Color-based	Minimum	Less	Potey et al. [156], Jain et al. [157]
Random password	Maximum	Less	Alhothaily et al. [30], Prabhu and Shah [131], Hassan et al. [163]
Augmented	Maximum	High	L’Yi et al. [170], Seo et al. [171], Zhang et al. [172]

## Abbreviations

The following abbreviations are used in this manuscript:

MPAS	Mobile Password Authentication Schemes
PIN	Personal Identification Number
EEG	Electroencephalography
ECG	Electrocardiogram
SIM	Subscriber Identity Module
KNN	K-nearest Neighbor
SVM	Support Vector Machine
CNN	Convolutional Neural Network
DTW	Dynamic Time Warping
LSTM	Long Short-term Memory
RNN	Recurrent Neural Network
BN	Bayesian Network
CA	Continuous Authentication
EER	Equal Error Rate
CVS	Continuous Verification System

## References

[1] Khan W.Z., Xiang Y., Aalsalem M.Y., Arshad Q. (2012). Mobile phone sensing systems: A survey. IEEE Commun. Surv. Tutor..

[2] Goyal A., Matta P., Lohumi Y. (2024). Preventing Shoulder Surfing Attacks_Matrix Based Graphical Technique. Proceedings of the 2024 IEEE International Conference for Women in Innovation, Technology & Entrepreneurship (ICWITE).

[3] Patrick A.S., Long A.C., Flinn S. HCI and security systems. Proceedings of the CHI ’03 Extended Abstracts on Human Factors in Computing Systems—CHI ’03.

[4] Zhang Z., Ning H., Shi F., Farha F., Xu Y., Xu J., Zhang F., Choo K.K.R. (2022). Artificial intelligence in cyber security: Research advances, challenges, and opportunities. Artif. Intell. Rev..

[5] Said G., Ghani A., Ullah A., Azeem M., Bilal M., Kwak K.S. (2022). Light-weight secure aggregated data sharing in IoT-enabled wireless sensor networks. IEEE Access.

[6] Wang C., Wang Y., Chen Y., Liu H., Liu J. (2020). User authentication on mobile devices: Approaches, threats and trends. Comput. Netw..

[7] Ghani A., Mansoor K., Mehmood S., Chaudhry S.A., Rahman A.U., Najmus Saqib M. (2019). Security and key management in IoT-based wireless sensor networks: An authentication protocol using symmetric key. Int. J. Commun. Syst..

[8] Dasgupta D., Nag A.K., Roy A. (2018). Adaptive Multi-Factor Authentication System. U.S. Patent.

[9] Mobile Marketing Statistics Compilation. https://www.smartinsights.com/mobile-marketing/mobile-marketing-analytics/mobile-marketing-statistics/.

[10] Tsoukas V., Gkogkidis A., Kakarountas A. A Survey on Mobile User Perceptions of Sensitive Data and Authentication Methods. Proceedings of the 24th Pan-Hellenic Conference on Informatics, PCI 2020.

[11] Kumar S.A., Ramya R., Rashika R., Renu R. (2021). A Survey on Graphical Authentication System Resisting Shoulder Surfing Attack. Advances in Artificial Intelligence and Data Engineering.

[12] Gilhooly K. (2005). Biometrics: Getting back to business. Comput. May.

[13] Li Y., Yun X., Fang L., Ge C. (2021). An Efficient Login Authentication System against Multiple Attacks in Mobile Devices. Symmetry.

[14] Kunda D., Chishimba M. (2021). A survey of android mobile phone authentication schemes. Mob. Netw. Appl..

[15] Patel S.S., Jaiswal A., Arora Y., Sharma B. (2021). Survey on Graphical Password Authentication System. Data Intell. Cogn. Inform..

[16] Manisha, Kumar N. (2020). Cancelable Biometrics: A comprehensive survey. Art. Intell. Rev..

[17] Rui Z., Yan Z. (2018). A survey on biometric authentication: Toward secure and privacy-preserving identification. IEEE Access.

[18] Ometov A., Bezzateev S., Mäkitalo N., Andreev S., Mikkonen T., Koucheryavy Y. (2018). Multi-factor authentication: A survey. Cryptography.

[19] Arias-Cabarcos P., Krupitzer C., Becker C. (2019). A survey on Adaptive Authentication. ACM Comput. Surv. (CSUR).

[20] Aris H., Yaakob W.F. (2018). Shoulder surf resistant screen locking for smartphones: A review of fifty non-biometric methods. Proceedings of the 2018 IEEE Conference on Application, Information and Network Security (AINS).

[21] Yuan E., Esfahani N., Malek S. (2014). A systematic survey of self-protecting software systems. ACM Trans. Auton. Adapt. Syst. (TAAS).

[22] Tziakouris G., Bahsoon R., Babar M.A. (2018). A survey on self-adaptive security for large-scale open environments. ACM Comput. Surv. (CSUR).

[23] Rittenhouse R., Chaudhry J. A survey of alternative authentication methods. Proceedings of the International Conference on Recent Advances in Computer Systems.

[24] Ferrag M.A., Maglaras L., Derhab A., Janicke H. (2020). Authentication schemes for smart mobile devices: Threat models, countermeasures, and open research issues. Telecommun. Syst..

[25] Ali G., Ally Dida M., Elikana Sam A. (2020). Two-factor authentication scheme for mobile money: A review of threat models and countermeasures. Future Internet.

[26] Alotaibi S., Furnell S., Clarke N. Transparent authentication systems for mobile device security: A review. Proceedings of the 2015 10th International Conference for Internet Technology and Secured Transactions (ICITST).

[27] Khan I., Ghani A., Saqlain S.M., Ashraf M.U., Alzahrani A., Kim D.H. (2023). Secure Medical Data Against Unauthorized Access using Decoy Technology in Distributed Edge Computing Networks. IEEE Access.

[28] Yampolskiy R.V. User Authentication via Behavior Based Passwords. Proceedings of the 2007 IEEE Long Island Systems, Applications and Technology Conference.

[29] Eiband M., Khamis M., von Zezschwitz E., Hussmann H., Alt F. Understanding Shoulder Surfing in the Wild. Proceedings of the 2017 CHI Conference on Human Factors in Computing Systems—CHI ’17.

[30] Alhothaily A., Hu C., Alrawais A., Song T., Cheng X., Chen D. (2017). A Secure and Practical Authentication Scheme Using Personal Devices. IEEE Access.

[31] Muslukhov I., Boshmaf Y., Kuo C., Lester J., Beznosov K. Know your enemy. Proceedings of the 15th International Conference on Human-Computer Interaction with Mobile Devices and Services—MobileHCI ’13.

[32] Corbett M., David-John B., Shang J., Ji B. (2024). ShouldAR: Detecting Shoulder Surfing Attacks Using Multimodal Eye Tracking and Augmented Reality. Proc. ACM Interact. Mob. Wearable Ubiquitous Technol..

[33] Chen Y., Yu Y., Zhai L. InfinityGauntlet: Brute-force attack on smartphone fingerprint authentication. Proceedings of the 32nd USENIX Conference on Security Symposium.

[34] Narayanan A., Shmatikov V. Fast dictionary attacks on passwords using time-space tradeoff. Proceedings of the 12th ACM conference on Computer and communications security—CCS ’05.

[35] Bosnjak L., Sres J., Brumen B. Brute-force and dictionary attack on hashed real-world passwords. Proceedings of the 2018 41st International Convention on Information and Communication Technology, Electronics and Microelectronics (MIPRO).

[36] Alkhwaja I., Albugami M., Alkhwaja A., Alghamdi M., Abahussain H., Alfawaz F., Almurayh A., Min-Allah N. (2023). Password cracking with brute force algorithm and dictionary attack using parallel programming. Appl. Sci..

[37] Syverson P. A taxonomy of replay attacks [cryptographic protocols]. Proceedings of the Computer Security Foundations Workshop VII.

[38] Li J. Design of authentication protocols preventing replay attacks. Proceedings of the 2009 International Conference on Future BioMedical Information Engineering (FBIE).

[39] Mehmood Z., Ch S.A., Nasar W., Ghani A. An efficient key agreement with rekeying for secured body sensor networks. Proceedings of the 2012 Second International Conference on Digital Information Processing and Communications (ICDIPC).

[40] Uusitalo I., Catot J.M., Loureiro R. Phishing and Countermeasures in Spanish Online Banking. Proceedings of the 2009 Third International Conference on Emerging Security Information, Systems and Technologies.

[41] Kirda E., Kruegel C. Protecting Users Against Phishing Attacks with AntiPhish. Proceedings of the 29th Annual International Computer Software and Applications Conference (COMPSAC’05).

[42] Baig M.M., Mahmood W. A Robust Technique of Anti Key-Logging using Key-Logging Mechanism. Proceedings of the 2007 Inaugural IEEE-IES Digital EcoSystems and Technologies Conference.

[43] Raza M., Iqbal M., Sharif M., Haider W. (2012). A survey of password attacks and comparative analysis on methods for secure authentication. World Appl. Sci. J..

[44] Biddle R., Chiasson S., Oorschot P.V. (2012). Graphical passwords. ACM Comput. Surv..

[45] Tang Y., Chen Y., Luo Y., Dong S., Li T. (2023). VR-PEKS: A Verifiable and Resistant to Keyword Guess Attack Public Key Encryption with Keyword Search Scheme. Appl. Sci..

[46] Pinkas B., Sander T. Securing passwords against dictionary attacks. Proceedings of the 9th ACM Conference on Computer and Communications Security—CCS ’02.

[47] Kwon T., Na S. (2014). TinyLock: Affordable defense against smudge attacks on smartphone pattern lock systems. Comput. Secur..

[48] Cha S., Kwag S., Kim H., Huh J.H. Boosting the Guessing Attack Performance on Android Lock Patterns with Smudge Attacks. Proceedings of the 2017 ACM on Asia Conference on Computer and Communications Security.

[49] Kumar P., Saini R., Roy P.P., Dogra D.P. (2017). A bio-signal based framework to secure mobile devices. J. Netw. Comput. Appl..

[50] Salama G.M., El-Gazar S., Omar B., Hassan A. (2024). Multimodal cancelable biometric authentication system based on EEG signal for IoT applications. J. Opt..

[51] Fujita K., Hirakawa Y. A study of password authentication method against observing attacks. Proceedings of the 2008 6th International Symposium on Intelligent Systems and Informatics.

[52] Patel J., Patel A. (2015). A Survey on Different Authentication Schemes for Session Passwords. Int. J. Sci. Res. Sci. Eng. Technol..

[53] Mayrhofer R., Sigg S. (2021). Adversary models for mobile device authentication. ACM Comput. Surv. (CSUR).

[54] Aslam M.U., Derhab A., Saleem K., Abbas H., Orgun M., Iqbal W., Aslam B. (2017). A survey of authentication schemes in telecare medicine information systems. J. Med. Syst..

[55] Velásquez I., Caro A., Rodríguez A. (2018). Authentication schemes and methods: A systematic literature review. Inf. Softw. Technol..

[56] Kilinc H.H., Yanik T. (2013). A survey of SIP authentication and key agreement schemes. IEEE Commun. Surv. Tutor..

[57] Faruki P., Bharmal A., Laxmi V., Ganmoor V., Gaur M.S., Conti M., Rajarajan M. (2014). Android security: A survey of issues, malware penetration, and defenses. IEEE Commun. Surv. Tutor..

[58] Meng W., Wong D.S., Furnell S., Zhou J. (2014). Surveying the development of biometric user authentication on mobile phones. IEEE Commun. Surv. Tutor..

[59] Teh P.S., Zhang N., Teoh A.B.J., Chen K. (2016). A survey on touch dynamics authentication in mobile devices. Comput. Secur..

[60] Sharma A., Mishra S. A Security Analysis of Password Managers on Android. Proceedings of the International Conference on Information Systems Security.

[61] Forget A. (2013). A World with Many Authentication Schemes. Ph.D. Thesis.

[62] Bátiz-Lazo B., Reid R. (2010). The development of cash-dispensing technology in the UK. IEEE Ann. Hist. Comput..

[63] Bonneau J., Preibusch S., Anderson R. A birthday present every eleven wallets? The security of customer-chosen banking pins. Proceedings of the International Conference on Financial Cryptography and Data Security.

[64] Staneková L., Stanek M. (2013). Analysis of dictionary methods for PIN selection. Comput. Secur..

[65] Binbeshr F., Por L.Y., Kiah M.L.M., Zaidan A.A., Imam M. (2023). Secure PIN-Entry Method Using One-Time PIN (OTP). IEEE Access.

[66] Van Nguyen T., Sae-Bae N., Memon N. (2017). DRAW-A-PIN: Authentication using finger-drawn PIN on touch devices. Comput. Secur..

[67] Ku Y., Park L.H., Shin S., Kwon T. (2019). Draw it as shown: Behavioral pattern lock for mobile user authentication. IEEE Access.

[68] Ye G., Tang Z., Fang D., Chen X., Wolff W., Aviv A.J., Wang Z. (2018). A Video-Based Attack for Android Pattern Lock. ACM Trans. Priv. Secur..

[69] Alajmi M., Elashry I., El-Sayed H.S., Faragallah O.S. (2020). A Password-Based Authentication System Based on the CAPTCHA AI Problem. IEEE Access.

[70] Wang C., Tang H., Zhu H., Zheng J., Jiang C. (2024). Behavioral authentication for security and safety. Secur. Saf..

[71] Li L., Zhao X., Xue G. Unobservable Re-authentication for Smartphones. Proceedings of the NDSS.

[72] Frank M., Biedert R., Ma E., Martinovic I., Song D. (2013). Touchalytics: On the Applicability of Touchscreen Input as a Behavioral Biometric for Continuous Authentication. IEEE Trans. Inf. Forensics Secur..

[73] Xu H., Zhou Y., Lyu M.R. Towards Continuous and Passive Authentication via Touch Biometrics: An Experimental Study on Smartphones. Proceedings of the 10th Symposium On Usable Privacy and Security (SOUPS 2014).

[74] Wang H., Chen T., Liu X., Chen J. (2020). Exploring the Hand and Finger-Issued Behaviors Toward Natural Authentication. IEEE Access.

[75] Fridman L., Weber S., Greenstadt R., Kam M. (2017). Active Authentication on Mobile Devices via Stylometry, Application Usage, Web Browsing, and GPS Location. IEEE Syst. J..

[76] Ul hassan S.S., Ghani A., Bilal M., Jolfaei A. (2021). Multi-Factor Pattern Implicit Authentication. IEEE Consum. Electron. Mag..

[77] Sudhakar T., Gavrilova M. (2020). Deep Learning for Multi-Instance Biometric Privacy. ACM Trans. Manage. Inf. Syst..

[78] Dargan S., Kumar M. (2020). A comprehensive survey on the biometric recognition systems based on physiological and behavioral modalities. Expert Syst. Appl..

[79] Al Abdulwahid A., Clarke N., Stengel I., Furnell S., Reich C. (2016). Continuous and transparent multimodal authentication: Reviewing the state of the art. Clust. Comput..

[80] Ryu R., Yeom S., Kim S.H., Herbert D. (2021). Continuous Multimodal Biometric Authentication Schemes: A Systematic Review. IEEE Access.

[81] Al-Garadi M.A., Mohamed A., Al-Ali A.K., Du X., Ali I., Guizani M. (2020). A survey of machine and deep learning methods for internet of things (IoT) security. IEEE Commun. Surv. Tutor..

[82] Bo C., Zhang L., Li X.Y., Huang Q., Wang Y. Silentsense: Silent user identification via touch and movement behavioral biometrics. Proceedings of the 19th Annual International Conference on Mobile Computing & Networking.

[83] Song Y., Cai Z., Zhang Z.L. Multi-touch authentication using hand geometry and behavioral information. Proceedings of the 2017 IEEE Symposium on Security and Privacy (SP).

[84] Ehatisham-ul Haq M., Azam M.A., Naeem U., ur Rehman S., Khalid A. (2017). Identifying smartphone users based on their activity patterns via mobile sensing. Procedia Comput. Sci..

[85] Liang Y., Cai Z., Yu J., Han Q., Li Y. (2018). Deep learning based inference of private information using embedded sensors in smart devices. IEEE Netw..

[86] Sajjad M., Khan S., Hussain T., Muhammad K., Sangaiah A.K., Castiglione A., Esposito C., Baik S.W. (2019). CNN-based anti-spoofing two-tier multi-factor authentication system. Pattern Recognit. Lett..

[87] Li W., Wang Y., Li J., Xiang Y. (2020). Toward supervised shape-based behavioral authentication on smartphones. J. Inf. Secur. Appl..

[88] Stragapede G., Vera-Rodriguez R., Tolosana R., Morales A., Acien A., Le Lan G. (2022). Mobile behavioral biometrics for passive authentication. Pattern Recognit. Lett..

[89] Stragapede G., Vera-Rodriguez R., Tolosana R., Morales A. (2023). BehavePassDB: Public database for mobile behavioral biometrics and benchmark evaluation. Pattern Recognit..

[90] Sejjari A., Moujahdi C., Assad N., Abdelfatteh H. (2024). Dynamic authentication on mobile devices: Evaluating continuous identity verification through swiping gestures. Signal Image Video Proc..

[91] Bello A.A., Chiroma H., Gital A.Y., Gabralla L.A., Abdulhamid S.M., Shuib L. (2020). Machine learning algorithms for improving security on touch screen devices: A survey, challenges and new perspectives. Neural Comput. Appl..

[92] Saini B.S., Singh P., Nayyar A., Kaur N., Bhatia K.S., El-Sappagh S., Hu J. (2020). A Three-Step Authentication Model for Mobile Phone User Using Keystroke Dynamics. IEEE Access.

[93] Sun Y., Upadhyaya S. Synthetic Forgery Attack against Continuous Keystroke Authentication Systems. Proceedings of the 2018 27th International Conference on Computer Communication and Networks (ICCCN).

[94] Kambourakis G., Damopoulos D., Papamartzivanos D., Pavlidakis E. (2014). Introducing touchstroke: Keystroke-based authentication system for smartphones. Secur. Commun. Netw..

[95] Khan H., Hengartner U., Vogel D. (2020). Mimicry attacks on smartphone keystroke authentication. ACM Trans. Priv. Secur. (TOPS).

[96] Buschek D., De Luca A., Alt F. Improving Accuracy, Applicability and Usability of Keystroke Biometrics on Mobile Touchscreen Devices. Proceedings of the 33rd Annual ACM Conference on Human Factors in Computing Systems, CHI ’15.

[97] Buchoux A., Clarke N. (2008). Deployment of Keystroke Analysis on a Smartphone. Proceedings of the Australian Information Security Management Conference.

[98] Saevanee H., Bhattarakosol P. Authenticating User Using Keystroke Dynamics and Finger Pressure. Proceedings of the 2009 6th IEEE Consumer Communications and Networking Conference.

[99] Zahid S., Shahzad M., Khayam S.A., Farooq M. (2009). Keystroke-Based User Identification on Smart Phones. Lecture Notes in Computer Science.

[100] seob Hwang S., Cho S., Park S. (2009). Keystroke dynamics-based authentication for mobile devices. Comput. Secur..

[101] Baig A.F., Eskeland S. (2021). Security, Privacy, and Usability in Continuous Authentication: A Survey. Sensors.

[102] Teh P.S., Teoh A.B.J., Yue S. (2013). A survey of keystroke dynamics biometrics. Sci. World J..

[103] Li F., Clarke N., Papadaki M., Dowland P. Behaviour Profiling for Transparent Authentication for Mobile Devices. Proceedings of the 10th European Conference on Information Warfare and Security.

[104] Chen J., Zhu G., Yang J., Jing Q., Bai P., Yang W., Qi X., Su Y., Wang Z.L. (2015). Personalized keystroke dynamics for self-powered human–machine interfacing. ACS Nano.

[105] Shi D., Tao D., Wang J., Yao M., Wang Z., Chen H., Helal S. (2021). Fine-Grained and Context-Aware Behavioral Biometrics for Pattern Lock on Smartphones. Proc. ACM Interact. Mob. Wearable Ubiquitous Technol..

[106] Colley A., Seitz T., Lappalainen T., Kranz M., Häkkilä J. (2016). Extending the Touchscreen Pattern Lock Mechanism with Duplicated and Temporal Codes. Adv. Hum.-Comp. Int..

[107] Eiband M., von Zezschwitz E., Buschek D., Hußmann H. My Scrawl Hides It All. Proceedings of the 2016 CHI Conference Extended Abstracts on Human Factors in Computing Systems—CHI EA ’16.

[108] Krombholz K., Hupperich T., Holz T. (2017). May the Force Be with You: The Future of Force-Sensitive Authentication. IEEE Internet Comput..

[109] Chakraborty N., Randhawa G.S., Das K., Mondal S. (2016). MobSecure: A Shoulder Surfing Safe Login Approach Implemented on Mobile Device. Procedia Comput. Sci..

[110] Vaddepalli S., Nivas S., Chettoor Jayakrishnan G., Sirigireddy G., Banahatti V., Lodha S. PassO–New Circular Patter Lock Scheme Evaluation. Proceedings of the 22nd International Conference on Human-Computer Interaction with Mobile Devices and Services.

[111] Gattulli V., Impedovo D., Pirlo G., Volpe F. (2023). Touch events and human activities for continuous authentication via smartphone. Sci. Rep..

[112] Finnegan O., White III J., Armstrong B., Adams E., Burkart S., Beets M., Nelakuditi S., Willis E., von Klinggraeff L., Parker H. (2024). The utility of behavioral biometrics in user authentication and demographic characteristic detection: A scoping review. Syst. Rev..

[113] Özbek M.E., Haytom M.A., Cherrier E. Recognition of biometric unlock pattern by GMM-UBM. Proceedings of the 2018 26th Signal Processing and Communications Applications Conference (SIU).

[114] Bošnjak L., Brumen B. (2020). Shoulder surfing experiments: A systematic literature review. Comput. Secur..

[115] Huang A., Gao S., Chen J., Xu L., Nathan A. (2020). High security user authentication enabled by piezoelectric keystroke dynamics and machine learning. IEEE Sens. J..

[116] Fang L., Li Y., Yun X., Wen Z., Ji S., Meng W., Cao Z., Tanveer M. (2019). THP: A novel authentication scheme to prevent multiple attacks in SDN-based IoT network. IEEE Internet Things J..

[117] Abdrabou Y., Pfeuffer K., Khamis M., Alt F. GazeLockPatterns: Comparing Authentication Using Gaze and Touch for Entering Lock Patterns. Proceedings of the ACM Symposium on Eye Tracking Research and Applications.

[118] Li Y., Cao Z., Wang J. (2017). Gazture. Proc. ACM Interact. Mob. Wearable Ubiquitous Technol..

[119] Rajanna V., Hammond T. GAWSCHI. Proceedings of the Ninth Biennial ACM Symposium on Eye Tracking Research & Applications—ETRA ’16.

[120] Rajanna V., Malla A.H., Bhagat R.A., Hammond T. DyGazePass: A gaze gesture-based dynamic authentication system to counter shoulder surfing and video analysis attacks. Proceedings of the 2018 IEEE 4th International Conference on Identity, Security, and Behavior Analysis (ISBA).

[121] Khamis M., Alt F., Hassib M., von Zezschwitz E., Hasholzner R., Bulling A. GazeTouchPass. Proceedings of the 2016 CHI Conference Extended Abstracts on Human Factors in Computing Systems—CHI EA ’16.

[122] Katsini C., Raptis G.E., Fidas C., Avouris N. Towards gaze-based quantification of the security of graphical authentication schemes. Proceedings of the 2018 ACM Symposium on Eye Tracking Research & Applications—ETRA ’18.

[123] Abdrabou Y., Khamis M., Eisa R.M., Ismail S., Elmougy A. Just gaze and wave: Exploring the use of gaze and gestures for shoulder-surfing resilient authentication. Proceedings of the 11th ACM Symposium on Eye Tracking Research & Applications.

[124] Abe N., Yamada S. A Novel Quality Assessment Method for Eye Movement Authentication. Proceedings of the 2020 Asia-Pacific Signal and Information Processing Association Annual Summit and Conference (APSIPA ASC).

[125] Constantinides A., Belk M., Fidas C., Pitsillides A. An eye gaze-driven metric for estimating the strength of graphical passwords based on image hotspots. Proceedings of the 25th International Conference on Intelligent User Interfaces.

[126] Namnakani O., Sinrattanavong P., Abdrabou Y., Bulling A., Alt F., Khamis M. Gazecast: Using mobile devices to allow gaze-based interaction on public displays. Proceedings of the 2023 Symposium on Eye Tracking Research and Applications.

[127] Abdelrahman Y., Khamis M., Schneegass S., Alt F. Stay cool! understanding thermal attacks on mobile-based user authentication. Proceedings of the 2017 CHI Conference on Human Factors in Computing Systems.

[128] Katsini C., Abdrabou Y., Raptis G.E., Khamis M., Alt F. The role of eye gaze in security and privacy applications: Survey and future HCI research directions. Proceedings of the 2020 CHI Conference on Human Factors in Computing Systems.

[129] Peng R., Gao Y., Jin Z. (2024). Gazenum: Unlock your phone with gaze tracking viewing numbers for authentication. CCF Trans. Pervasive Comput. Interact..

[130] Kopácsi L., Schneider T.S., Karr C., Barz M., Sonntag D. GazeLock: Gaze-and Lock Pattern-Based Authentication. Proceedings of the 30th ACM Symposium on Virtual Reality Software and Technology.

[131] Prabhu S., Shah V. (2015). Authentication Using Session Based Passwords. Procedia Comput. Sci..

[132] Golar P.C., Khandelwal B. Study of Usability Parameter for Graphical Based Authentication System. Proceedings of the 2020 9th International Conference System Modeling and Advancement in Research Trends (SMART).

[133] Sarohi H.K., Khan F.U. (2013). Graphical password authentication schemes: Current status and key issues. Int. J. Comput. Sci. Issues (IJCSI).

[134] Tari F., Ozok A.A., Holden S.H. A comparison of perceived and real shoulder-surfing risks between alphanumeric and graphical passwords. Proceedings of the second symposium on Usable privacy and security—SOUPS ’06.

[135] Florencio D., Herley C. A large-scale study of web password habits. Proceedings of the 16th International Conference on World Wide Web—WWW ’07.

[136] Jermyn I., Mayer A., Monrose F., Reiter M.K., Rubin A.D. (1999). The design and analysis of graphical passwords. Proceedings of the 8th USENIX Security Symposium.

[137] Gao H., Jia W., Ye F., Ma L. (2013). A survey on the use of graphical passwords in security. JSW.

[138] Everitt K.M., Bragin T., Fogarty J., Kohno T. A comprehensive study of frequency, interference, and training of multiple graphical passwords. Proceedings of the 27th International Conference on Human Factors in Computing Systems—CHI 09.

[139] Vaddeti A., Vidiyala D., Puritipati V., Ponnuru R.B., Shin J.S., Alavalapati G.R. (2020). Graphical passwords: Behind the attainment of goals. Secur. Priv..

[140] Kayem A.V. Graphical Passwords—A Discussion. Proceedings of the 2016 30th International Conference on Advanced Information Networking and Applications Workshops (WAINA).

[141] Wiedenbeck S., Waters J., Birget J.C., Brodskiy A., Memon N. Authentication using graphical passwords: Effects of tolerance and image choice. Proceedings of the 2005 Symposium on Usable Privacy and Security.

[142] Luca A.D., Hang A., Brudy F., Lindner C., Hussmann H. Touch me once and i know it’s you!. Proceedings of the 2012 ACM Annual Conference on Human Factors in Computing Systems—CHI ’12.

[143] Sun H., Chen S., Yeh J., Cheng C. (2018). A Shoulder Surfing Resistant Graphical Authentication System. IEEE Trans. Dependable Secur. Comput..

[144] Wiedenbeck S., Waters J., Birget J.C., Brodskiy A., Memon N. (2005). PassPoints: Design and longitudinal evaluation of a graphical password system. Int. J. Hum.-Comput. Stud..

[145] Thorpe J., van Oorschot P.C. Human-seeded Attacks and Exploiting Hot-spots in Graphical Passwords. Proceedings of the 16th USENIX Security Symposium on USENIX Security Symposium, SS’07.

[146] van Oorschot P.C., Thorpe J. (2011). Exploiting Predictability in Click-based Graphical Passwords. J. Comput. Secur..

[147] Gołofit K. (2007). Click passwords under investigation. European Symposium on Research in Computer Security.

[148] Cain A.A., Still J.D. (2016). A Rapid Serial Visual Presentation Method for Graphical Authentication. Advances in Intelligent Systems and Computing.

[149] Wiedenbeck S., Waters J., Sobrado L., Birget J.C. Design and evaluation of a shoulder-surfing resistant graphical password scheme. Proceedings of the Working Conference on Advanced Visual Interfaces—AVI ’06.

[150] Yu Z., Olade I., Liang H.N., Fleming C. Usable Authentication Mechanisms for Mobile Devices: An Exploration of 3D Graphical Passwords. Proceedings of the 2016 International Conference on Platform Technology and Service (PlatCon).

[151] Irfan K., Anas A., Malik S., Amir S. Text based graphical password system to obscure shoulder surfing. Proceedings of the 2018 15th International Bhurban Conference on Applied Sciences and Technology (IBCAST).

[152] Khan M.A., Din I.U., Almogren A. (2023). Securing access to internet of medical things using a graphical-password-based user authentication scheme. Sustainability.

[153] Izadeen G.Y., Ameen S.Y. (2021). Smart android graphical password strategy: A review. Asian J. Res. Comput. Sci..

[154] Das S., Wang B., Tingle Z., Camp L.J. (2019). Evaluating user perception of multi-factor authentication: A systematic review. arXiv.

[155] Hong W., Chen J., Chang P.S., Wu J., Chen T.S., Lin J. (2021). A Color Image Authentication Scheme with Grayscale Invariance. IEEE Access.

[156] Potey M.M., Dhote C.A., Sharma D.H. Secure authentication for data protection in cloud computing using color schemes. Proceedings of the 2016 International Conference on Computation System and Information Technology for Sustainable Solutions (CSITSS).

[157] Jain A., Khetan R., Dubey K., Rambade H. (2017). Color Shuffling Password Based Authentication. Int. J..

[158] Chiang H.Y., Chiasson S. Improving user authentication on mobile devices: A touchscreen graphical password. Proceedings of the 15th International Conference on Human-Computer Interaction with Mobile Devices and Services.

[159] Gugenheimer J., De Luca A., Hess H., Karg S., Wolf D., Rukzio E. Colorsnakes: Using colored decoys to secure authentication in sensitive contexts. Proceedings of the 17th International Conference on Human-Computer Interaction with Mobile Devices and Services.

[160] Woods N., Silvennoinen J. (2023). Enhancing the user authentication process with colour memory cues. Behav. Inf. Technol..

[161] Selamat S.R., Cai S.Y., Hassan N.H., Yusof R. (2024). An Algorithm for Color-Based Password Authentication to Increase Security Level. Innov. Res. Inform. (INNOVATICS).

[162] Fong J., Poet R. Creating Graphical Passwords on a Mobile Phone: Graphical Passwords on a Mobile. Proceedings of the 13th International Conference on Security of Information and Networks.

[163] Hassan S.S., Ullah S., Afzal S., Khan M.A., Khan M.A., Akbar H. Servers Voice Graphical Authentication. Proceedings of the 2015 12th International Conference on Fuzzy Systems and Knowledge Discovery (FSKD).

[164] Naqvi S.S.u.H., Afzal S. Operation code authentication preventing shoulder surfing attacks. Proceedings of the 2010 3rd International Conference on Computer Science and Information Technology.

[165] Imtiaz N., Wahid A., Hasan S.S.U., Akbar H., Ahmed A. (2022). Behavioral Authentication for Smartphones backed by “Something you Process”. Found. Univ. J. Eng. Appl. Sci..

[166] Dan Y.X., Ku W.C. A simple observation attacks resistant PIN-entry scheme employing audios. Proceedings of the 2017 IEEE 9th International Conference on Communication Software and Networks (ICCSN).

[167] Hasan A., Qureshi K. Internet of things device authentication scheme using hardware serialization. Proceedings of the 2018 International Conference on Applied and Engineering Mathematics (ICAEM).

[168] Xu W., Liang H.N., Zhao Y., Yu D., Monteiro D. DMove: Directional motion-based interaction for augmented reality head-mounted displays. Proceedings of the 2019 CHI Conference on Human Factors in Computing Systems.

[169] Yu D., Liang H.N., Lu X., Zhang T., Xu W. Depthmove: Leveraging head motions in the depth dimension to interact with virtual reality head-worn displays. Proceedings of the 2019 IEEE International Symposium on Mixed and Augmented Reality (ISMAR).

[170] L’Yi S., Koh K., Jo J., Kim B., Seo J. CloakingNote. Proceedings of the 29th Annual Symposium on User Interface Software and Technology—UIST ’16.

[171] Seo H., Kim J., Kim H., Liu Z. (2017). Personal identification number entry for Google glass. Comput. Electr. Eng..

[172] Zhang R., Zhang N., Du C., Lou W., Hou Y.T., Kawamoto Y. AugAuth: Shoulder-surfing resistant authentication for augmented reality. Proceedings of the 2017 IEEE International Conference on Communications (ICC).

[173] Olade I., Fleming C., Liang H.N. (2020). BioMove: Biometric User Identification from Human Kinesiological Movements for Virtual Reality Systems. Sensors.

[174] Corbett M., Shang J., Ji B. (2023). Gazepair: Efficient pairing of augmented reality devices using gaze tracking. IEEE Trans. Mob. Comput..

[175] Park S., Ha J., Kim L. (2024). Event-related pupillary response-based authentication system using eye-tracker add-on augmented reality glasses for individual identification. Front. Physiol..

[176] Miller R., Banerjee N.K., Banerjee S. Within-system and cross-system behavior-based biometric authentication in virtual reality. Proceedings of the 2020 IEEE Conference on Virtual Reality and 3D User Interfaces Abstracts and Workshops (VRW).

[177] Pahuja S., Goel N. (2024). Multimodal biometric authentication: A review. AI Commun..

[178] Bhattacharyya D., Ranjan R., Alisherov F., Choi M. (2009). Biometric authentication: A review. Int. J. U-E-Serv. Sci. Technol..

[179] Nguyen H.T., Nguyen L.T. (2019). Fingerprints classification through image analysis and machine learning method. Algorithms.

[180] Kumar M., Priyanka (2019). Various image enhancement and matching techniques used for fingerprint recognition system. Int. J. Inf. Technol..

[181] Chen X.Z., Lin J.L., Chen Y.L. Fod enroll image quality classification method for fingerprint authentication system. Proceedings of the 2021 International Symposium on Intelligent Signal Processing and Communication Systems (ISPACS).

[182] Sun H., Zhang Y., Chen P., Wang H., Liu Y.P., Liang R. (2023). A new approach in automated fingerprint presentation attack detection using optical coherence tomography. IEEE Trans. Inf. Forensics. Sec..

[183] Kortli Y., Jridi M., Al Falou A., Atri M. (2020). Face recognition systems: A survey. Sensors.

[184] Li Z., Yin B., Yao T., Guo J., Ding S., Chen S., Liu C. Sibling-attack: Rethinking transferable adversarial attacks against face recognition. Proceedings of the IEEE/CVF Conference on Computer Vision and Pattern Recognition.

[185] Dang T.V. (2023). Smart attendance system based on improved facial recognition. J. Robot. Control (JRC).

[186] Opanasenko V.M., Fazilov S.K., Mirzaev O.N., Sa’dullo ugli Kakharov S. (2024). An Ensemble Approach To Face Recognition In Access Control Systems. J. Mob. Multimed..

[187] Chaterjee U., Mukhopadhyay D., Chakraborty R.S. (2020). 3PAA: A private PUF protocol for anonymous authentication. IEEE Trans. Inf. Forensics Secur..

[188] Pathak A., Patil T., Pawar S., Raut P., Khairnar S. Secure authentication using zero knowledge proof. Proceedings of the 2021 Asian Conference on Innovation in Technology (ASIANCON).

[189] Hamouda B.E.H.H. (2020). Comparative study of different cryptographic algorithms. J. Inf. Secur..

[190] Garg S., Goel A., Jain A., Policharla G.V., Sekar S. zkSaaS: Zero-Knowledge SNARKs as a Service. Proceedings of the 32nd USENIX Security Symposium (USENIX Security 23).

[191] Baldimtsi F., Chalkias K.K., Ji Y., Lindstrøm J., Maram D., Riva B., Roy A., Sedaghat M., Wang J. (2024). zklogin: Privacy-preserving blockchain authentication with existing credentials. arXiv.

[192] Indushree M., Raj M., Mishra V.K., Shashidhara R., Das A.K., Bhat V. (2023). Mobile-Chain: Secure blockchain based decentralized authentication system for global roaming in mobility networks. Comput. Commun..

[193] Chaudhary D., Dadsena P.K., Padmavathi A., Hassan M.M., Alkhamees B.F., Kumar U. (2024). Anonymous Quantum Safe Construction of Three Party Authentication and Key Agreement Protocol for Mobile Devices. IEEE Access.

[194] Dee T., Richardson I., Tyagi A. Continuous transparent mobile device touchscreen soft keyboard biometric authentication. Proceedings of the 2019 32nd International Conference on Vlsi Design and 2019 18th International Conference on Embedded Systems (Vlsid).

[195] Gunn D.J., Roy K., Bryant K. Simulated cloud authentication based on touch dynamics with SVM. Proceedings of the 2018 IEEE Symposium Series on Computational Intelligence (SSCI).

[196] Sitová Z., Šeděnka J., Yang Q., Peng G., Zhou G., Gasti P., Balagani K.S. (2015). HMOG: New behavioral biometric features for continuous authentication of smartphone users. IEEE Trans. Inf. Forensics Secur..

[197] Eagle N., Pentland A. (2006). Reality mining: Sensing complex social systems. Pers. Ubiquitous Comput..

[198] Northcutt C., Jiang L., Chuang I. (2021). Confident learning: Estimating uncertainty in dataset labels. J. Artif. Intell. Res..

[199] Rosli M.M., Tempero E., Luxton-Reilly A. (2018). Evaluating the quality of datasets in software engineering. Adv. Sci. Lett..

[200] Schott L., Rauber J., Bethge M., Brendel W. (2018). Towards the first adversarially robust neural network model on MNIST. arXiv.

[201] Prabhu V.U. (2019). Kannada-MNIST: A new handwritten digits dataset for the Kannada language. arXiv.

[202] Tolosana R., Vera-Rodriguez R., Fierrez J., Ortega-Garcia J. Incorporating touch biometrics to mobile one-time passwords: Exploration of digits. Proceedings of the IEEE Conference on Computer Vision and Pattern Recognition Workshops.

[203] Tolosana R., Vera-Rodriguez R., Fierrez J., Ortega-Garcia J. (2020). BioTouchPass2: Touchscreen Password Biometrics Using Time-Aligned Recurrent Neural Networks. IEEE Trans. Inf. Forensics Secur..

[204] Acien A., Morales A., Fierrez J., Vera-Rodriguez R., Delgado-Mohatar O. (2021). BeCAPTCHA: Behavioral bot detection using touchscreen and mobile sensors benchmarked on HuMIdb. Eng. Appl. Artif. Intell..

[205] Han J.K., Bi X., Kim H., Woo S.S. PassTag: A Graphical-Textual Hybrid Fallback Authentication System. Proceedings of the 15th ACM Asia Conference on Computer and Communications Security.

[206] Mayrhofer R., Mohan V., Sigg S. (2020). Adversary Models for Mobile Device Authentication. arXiv.

[207] Wazir W., Khattak H.A., Almogren A., Khan M.A., Din I.U. (2020). Doodle-Based Authentication Technique Using Augmented Reality. IEEE Access.

[208] Badshah A., Ghani A., Daud A., Jalal A., Bilal M., Crowcroft J. (2023). Towards smart education through internet of things: A survey. ACM Comput. Surv..

[209] Khan B.U.I., Olanrewaju R.F., Anwar F., Mir R.N., Yaacob M. (2020). Scrutinising internet banking security solutions. Int. J. Inf. Comput. Secur..

[210] Irshad A., Chaudhry S.A., Ghani A., Mallah G.A., Bilal M., Alzahrani B.A. (2022). A low-cost privacy preserving user access in mobile edge computing framework. Comput. Electr. Eng..

[211] Kamra S., Scott J. (2019). Impact of Data Breaches to Organizations and Individuals. https://www.eccu.edu/blog/technology/data-breaches-threats-and-consequences/.

[212] Che Z., Wang Y., Zhao J., Qiang Y., Ma Y., Liu J. (2019). A distributed energy trading authentication mechanism based on a consortium blockchain. Energies.

[213] Jan S.U., Ghani A., Alzahrani A., Saqlain S.M., Yahya K., Sajjad H. (2023). Bandwidth and power efficient lightweight authentication scheme for healthcare system. J. King Saud Univ.-Comput. Inf. Sci..

[214] Roy A., Razia S., Parveen N., Rao A.S., Nayak S.R., Poonia R.C. (2020). Fuzzy rule based intelligent system for user authentication based on user behaviour. J. Discret. Math. Sci. Cryptogr..

[215] Croce F., Hein M. Reliable evaluation of adversarial robustness with an ensemble of diverse parameter-free attacks. Proceedings of the International Conference on Machine Learning.

[216] Harbach M., De Luca A., Egelman S. The anatomy of smartphone unlocking: A field study of android lock screens. Proceedings of the 2016 CHI Conference on Human Factors in Computing Systems.

[217] Yang J., Zhang X. (2012). Feature-level fusion of fingerprint and finger-vein for personal identification. Pattern Recognit. Lett..

[218] Zhang Q., Yin Y., Zhan D.C., Peng J. (2014). A Novel Serial Multimodal Biometrics Framework Based on Semisupervised Learning Techniques. IEEE Trans. Inf. Forensics Secur..

[219] Gupta P., Gupta P. (2018). Multibiometric Authentication System Using Slap Fingerprints, Palm Dorsal Vein, and Hand Geometry. IEEE Trans. Ind. Electron..

